# Public health emergency accelerated research response—the Clinical and Translational Science Institute of Southeast Wisconsin COVID-19 research initiative

**DOI:** 10.3389/fpubh.2025.1529121

**Published:** 2025-05-09

**Authors:** Michael P. Anello, Doriel D. Ward, Orsolya M. Garrison, Amit Gode, Octavian C. Ioachimescu, David R. Friedland, Reza Shaker

**Affiliations:** ^1^Clinical and Translational Science Institute of Southeast Wisconsin, The Medical College of Wisconsin, Milwaukee, WI, United States; ^2^Clement J. Zablocki Veteran Affairs Medical Center, Milwaukee, WI, United States; ^3^Department of Medicine, Division of Pulmonary, Critical Care, and Sleep Medicine, The Medical College of Wisconsin, Milwaukee, WI, United States; ^4^Department of Otolaryngology and Communication Sciences, The Medical College of Wisconsin, Milwaukee, WI, United States; ^5^Department of Medicine, Division of Gastroenterology and Hepatology, The Medical College of Wisconsin, Milwaukee, WI, United States

**Keywords:** clinical and translational science award, disaster medicine, ensemble, multidisciplinary collaboration, multi-institutional partnerships, team science and practice, translational and clinical research, translational workforce development

## Abstract

**Introduction:**

In March 2020, the National Center for Advancing Translational Sciences—Clinical and Translational Science Awards (CTSA) Program issued an urgent “Call to Action,” requesting CTSA hubs to accelerate clinical and translational research (C&TR) in response to the COVID-19 public health emergency. The Clinical and Translational Science Institute of Southeast Wisconsin (CTSI) quickly responded by launching a regional research initiative among its eight academic and healthcare partner institutions to nucleate teams around COVID-19 C&TR.

**Methods:**

A comprehensive search of COVID-19 funding opportunities, combined with suggestions from CTSI leadership and C&TR investigators, produced a list of 31 distinct C&TR questions that were used to nucleate investigators into teams. A survey was shared with the faculty of all eight partner institutions to solicit interest in joining the teams. Multidisciplinary team formation was based on a novel CTSI model, called the “Team Science-Guided Integrated Clinical and Research Ensemble (Ensemble).” In this model, teams are formed around an unmet patient medical need, based on the intentional recruitment of members from three domains: (1) the clinical and translational research enterprise, (2) the health care systems, and (3) the community of stakeholders. The teams were provided no funding, but received substantial CTSI research and administrative support.

**Results:**

Forty-one teams were formed, and 243 investigators participated during the first year of the initiative. Team efforts resulted in the submission of 21 grant proposals, totaling $32,528,297. Three grant proposals were funded, totaling $609,888. The research initiative generated eight publications and had a significant impact on patient health, involving a combined total of 456 research participants. The initiative led to several systemic improvements, by (1) exposing investigators to team science-guided C&TR (Ensembles), (2) increasing inter-institutional and inter-departmental collaborations, (3) creating new partnerships with community organizations, and (4) providing qualitative data on lessons learned.

**Conclusion:**

The COVID-19 regional research initiative provided a compelling model of how basic science, clinical/translational, and community researchers can be mobilized for accelerated C&TR to address a public health threat. The initiative demonstrated that the fundamentals of the novel CTSI Ensemble team concept can be leveraged to expedite the formation of highly efficient teams.

## 1 Introduction

The COVID-19 outbreak caught the world off guard but also underscored local opportunities to better prepare and quickly deploy emergency public health measures. Moreover, the early phase pressures of the pandemic magnified the common challenges of facilitating research collaboration between members of the translational research enterprise, health care systems, and community of stakeholders—entities that are oftentimes siloed, most notably under the acute strains of a crisis, e.g., the COVID-19 pandemic.

Like many academic health institutions, Froedtert Hospital & Medical College of Wisconsin (F&MCW) executed a swift response to the acute challenges of the COVID-19 pandemic by immediately focusing on patient and staff safety and addressing clinical and translational research (C&TR) priorities and policies. For example, in April 2020, the pandemic forced F&MCW to limit patient services to “essential-only” procedures, and on March 28^th^, 2020, all human subject research was suspended, except in cases where ceasing study activities could cause immediate and possibly life-threatening risks to subjects (1, 2). In addition, F&MCW research laboratories were hibernated (3) and laboratory PPE was donated to hospital staff to enhance patient and staff safety. Regardless of these challenges, F&MCW prioritized a path for the development of requisite C&TR and related treatments as a part of their overall strategy to address COVID-19. Investigators at F&MCW conducted a variety of COVID-19 research studies (4–12), many of these, focused on City of Milwaukee communities, which have been shown to experience significant health disparities (13–16). Research from these investigators focused on understanding COVID-19 transmission and health disparities in vulnerable Milwaukee populations and neighborhoods, as well as the social determinants of health relevant to these geographic locations (17–23).

At the national level, the response to the pandemic included a “Call to Action” issued by the National Institutes of Health (NIH), National Center for Advancing Translational Sciences (NCATS), Clinical and Translational Science Awards (CTSA) Program (24). The NCATS supports approximately 60 academic hubs across the country, most affiliated with health care systems. These CTSA hubs are committed to the goal of enhancing national capacity, methods, and processes in C&TR, and are focused on improving the health of local communities. Dr. Christopher P. Austin, Director of NCATS at the time, wrote in the March 31^st^, 2020, Director’s Corner:

We have turned our attention toward marshaling the amazing NCATS engine of innovation to overcome this pandemic. We are working closely with our CTSA Program grantees to rapidly share insights across major medical centers at various stages of the pandemic, swiftly initiate and support clinical research that will help determine how to detect and treat patients with COVID-19, and collaborate with our colleagues at the United States Food and Drug Administration, the Centers for Disease Control and Prevention, the Patient-Centered Outcomes Research Institute and elsewhere.

In response to this Call to Action, the Clinical and Translational Science Institute of Southeast Wisconsin (CTSI) immediately focused on developing a coordinated platform for C&TR. CTSI is a consortium of eight partner institutions that includes MCW, Froedtert Hospital, Children’s Wisconsin, Clement J. Zablocki Veterans Affairs Medical Center, Versiti Blood Center, Marquette University, University of Wisconsin-Milwaukee (UWM), and Milwaukee School of Engineering. The eight-member consortium operates as a borderless, synergistic metropolis in Southeast Wisconsin that leverages the strengths and expertise of each partner organization in addressing the healthcare needs of our communities. As such, the consortium was well-positioned to respond to the broad goals of the NCATS COVID-19 Call to Action.

This paper describes a first-of-its-kind, accelerated implementation of an inter-institutional collaborative regional infrastructure to support C&TR relevant to the COVID-19 pandemic. A critical component to the success of this research initiative, involved a novel concept for team formation and collaboration, called the “Team Science-Guided Integrated Clinical and Research Ensemble (Ensemble).” The Ensemble concept, developed in 2019 by Reza Shaker, MD, is designed to addresses the unmet health needs of patients and the community by assembling an interdisciplinary team that includes patients, community members, clinicians, as well as basic, clinical, and community engagement scientists.

The Ensemble concept draws heavily from the science of team science. Using the fundamentals of team science, Ensemble members develop solutions that can range from new diagnostic tools, treatments, or research proposals to new processes and procedures, e.g., new data science applications, new methods for vaccine development, public health safety measures, and community health programs. For the COVID-19 research initiative, the use of Ensemble fundamentals was critical to expedite the formation of highly productive teams. A description of these teams, their formation within a regional research initiative, successes, challenges, and lessons learned, are described in the sections, below.

Please note, this paper is not based on a standard C&TR study design, and the organization of the content reflects this distinction. The priority of the COVID-19 Research Initiative was to accelerate C&TR. It was not developed with traditional C&TR goals, e.g., to test a hypothesis, compare two different approaches, or study specific patient cohorts, etc. We used a narrative format to describe the “methodology” section because of the timeline’s importance to expediting the research initiative under pandemic conditions. What follows is a narrative review of the COVID-19 Research Initiative. The review discusses steps for team implementation; tracking of scholarly outcomes for the teams; case studies of team implementation and performance; observations of team science barriers with steps to address challenges; qualitative data to inform lessons learned; and discussion of the experience in terms of the literature on multidisciplinary team science.

## 2 COVID-19 research initiative: team implementation steps

On March 23^rd^, 2020, CTSI formed the COVID-19 Research Initiative Planning Committee (S1), comprised of 19 CTSI staff and faculty, to expedite COVID-19 research in Southeast Wisconsin. To obtain guidance from academic institutional leadership, CTSI formed the COVID-19 Research Initiative Leadership Advisory Committee (S2), comprised of 25 members from the CTSI Council of Collaboration, CTSI Executive Committee, and CTSI Ensemble Review Committee. These efforts led to a plan to leverage the regional infrastructure of CTSI to coalesce teams around COVID-19 research questions.

### 2.1 Team scope development

The initial scope of each team was predetermined through a process to identify important COVID-19 research questions. A list of questions was compiled from a comprehensive search on COVID-19 funding opportunities, and suggestions from leadership and investigators at CTSI partner institutions. This strategy had the dual purpose of (1) building teams around the most relevant research questions defined by local and national sources, and (2) providing teams with a greater chance of obtaining funding by beginning with research targets that already had funding mechanisms. Potential research questions were reviewed to (1) eliminate redundant or overlapping questions, (2) aggregate similar questions into broader themes or questions, and (3) remove questions without a C&TR goal. The resulting C&TR questions were used to nucleate investigators into focused research teams.

### 2.2 Invitation to participate on teams

The COVID-19 Research Initiative issued an invitation (S3) on April 7^th^, 2020, from the CTSI director and the dean of the MCW medical school to 2,000 faculty of MCW, and faculty at five other Southeastern Wisconsin health centers and academic institutions. The invitation explained the COVID-19 Research Initiative and invited interested faculty and staff to attend a town hall meeting. It also contained a hyperlink to a REDCap (25, 26) survey instrument that allowed faculty to join COVID-19 research teams.

### 2.3 Town hall meetings and early team implementation activities

On April 8^th^, 2020, two virtual COVID-19 Research Initiative town hall meetings were convened. CTSI leadership were joined by the Dean of the Medical School in an urgent appeal for faculty and staff to join the initiative. The agenda included an overview of the mission and responsibilities of a CTSA hub and the seamless structure and synergy of the eight-partner CTSI consortium. Methods for joining the initiative were explained, and investigators received their first COVID-19 team member assignment, to create a succinct one-slide/one-minute PowerPoint presentation describing their research experience and human and technical resources. These one-slide summaries were used to make introductions at initial team meetings. Participants were also provided an “investigator implementation roadmap (S4)” to explain the nucleation process for team formation. It contained early team implementation activities to expedite developing research ideas and identifying necessary resources and research supports. Attendees were provided information on research services led by CTSI and key contacts for further information. This was essential to route questions to appropriate individuals and keep information consistent. The town hall included novel fundamentals of team composition and operation that would be important to developing impactful patient solutions. These fundamentals were based on two models developed by CTSI; the Mutually Learning Tri-Lateral Ecosystem and the CTSI Team Science-Guided, Integrated Clinical and Research Ensemble.

### 2.4 Models for team composition and team process developed by CTSI

#### 2.4.1 Mutually learning tri-lateral ecosystem

**Figure 1:**
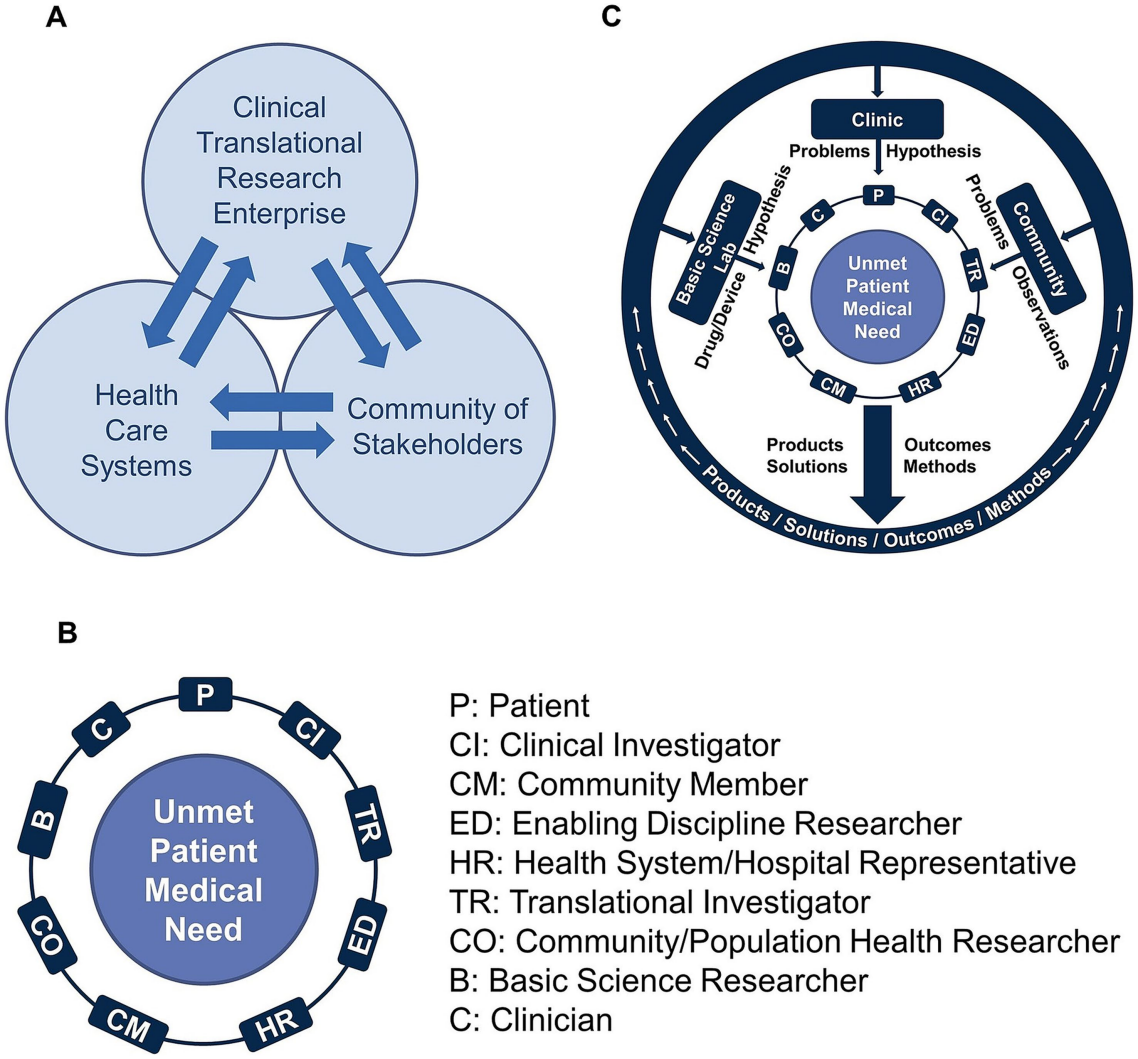
**(A)** The Mutually Learning Tri-lateral Ecosystem is a model of biomedical research that recognizes the existence of three domains: (1) clinical and translational enterprise; (2) health care systems; and (3) community of stakeholders. According to the model, barriers to effective biomedical research result from the siloed organization of these domains. **(B)** The Team Science-Guided, Integrated Clinical and Research Ensemble is the basic translational unit, the engine that drives the development of patient solutions. The team model is intentionally inclusive to connect what would otherwise remain as siloed stakeholders throughout the traditional research process. **(C)** Team members from the basic sciences, clinic, and community of stakeholders, bring forth problems, observations, and hypotheses, and create better solutions, because they do not originate through a siloed approach. Once the patient solutions are developed and implemented, there is a feedback mechanism facilitated by representatives of the three domains to provide long-term continuous review and refinement of the patient solutions.

The underlying fundamentals of the COVID-19 Research Initiative were based on a theoretical model of biomedical research called the “Mutually Learning Tri-lateral Ecosystem,” which was developed by CTSI over the past 15 years. The strategic model draws upon CTSI’s guiding principles of “All in Together” and “Achieving Together What We Cannot Achieve Alone.” Operationally, these core principles catalyze the participation of all those impacted by healthcare limitations and barriers, and those in positions to overcome such barriers in a multidisciplinary team science model. The Mutually Learning Tri-lateral Ecosystem describes the collaborative influence between three domains on the development and delivery of patient solutions. These domains are comprised of the (1) clinical and translational enterprise, (2) health care systems, and (3) community of stakeholders. CTSI has used this model to understand the barriers and influencers of biomedical research that result from the siloed organization of these domains. As seen in Figure 1A, the bidirectional arrows between the three domains indicate the potential for mutual learning and effecting while facilitating new synergies. This mutual learning includes sharing critical insights on unmet patient needs that can emanate from the basic science laboratory, the clinic, or the community while leveraging the expertise of clinical and translational researchers, supporting disciplines, relevant hospital representatives, and community of stakeholders and patients. Operationalization of this tri-lateral ecosystem led to the development of a novel model for team formation and C&TR processes, called the “Ensemble.” Together, these two frameworks provided the underlying fundamentals for teams in the COVID-19 Research Initiative.

#### 2.4.2 CTSI team science-guided integrated clinical and research ensemble

The Ensemble team model was developed to work directly within the Mutually Learning Tri-lateral Ecosystem as the basic translational research unit that drives the development of patient solutions. As shown in Figure 1B, a team is formed from diverse multidisciplinary Ensemble roles that come together around an unmet patient medical need. Importantly, patients, community members, and clinicians play a significant role, ensuring that solutions are both clinically relevant and practical. An Ensemble team is uniquely built to connect with all three domains of the Mutually Learning Tri-lateral Ecosystem (Figure 1C), such that the Ensemble has the benefit of perspectives and the exchange of ideas from all three domains. The Ensemble’s team structure provides a feedback loop and mechanism for sharing, disseminating, and implementing their findings and results, while also receiving feedback from communities, patients, researchers, and hospital representatives. An important fundamental of the Ensemble is for the team to remain adaptable throughout its existence, and continuously evolve to accommodate quickly changing research priorities that stem from Ensemble research results or external developments. Figure 1C illustrates the flow of ideas into the Ensemble, and the return of information, ideas, products, and solutions to the three domains. The Ensemble Program has been a cornerstone of the CTSI mission, dating back to the formation of the first Pre-Ensemble teams in June 2019, and the competitive review and funding of approved Ensembles in January 2020. With the debut of the CTSI Ensemble Program, the model was well-positioned for adaption to the COVID-19 Research Initiative.

##### 2.4.2.1 Team science is critical to ensemble success

The success of an Ensemble team is highly dependent on embracing the fundamentals of team science ─ a discipline that has gained greater attention in recent years as the need for multidisciplinary team collaboration continues to increase in numerous fields, including the biomedical sciences. Team science has been recognized as a critical approach to address complex problems. At the core of team science theory is the principle of interdependence, where investigators recognize that they need each other to reach goals they cannot achieve independently. Teams range in cross-disciplinary integration on a continuum that includes multidisciplinary, interdisciplinary, and transdisciplinary integration (25). In terms of team operations and culture, scholars have long acknowledged the importance of creating psychological safety (26–28) within the team, a concept introduced in 1999 by Edmondson, et al. (29) Psychological safety can be enhanced through a variety of facilitators, including the fundamentals of active listening, self-awareness, team-awareness, and trust (30, 31). The creation of psychological safety is also enabled by providing appropriate leadership style, promoting scientific disagreement among team members while containing personal conflict, and acknowledging the co-equality of ideas as team members develop a shared vision (30). A number of theoretical models have been developed to describe group development, but the model most recognized is Tuckman’s Model of Group Development (32). In fact, a literature search in 2010 found that the model was already cited in 1,740 articles (33), and a search of google scholars from 2015 to 2019 found over 20,000 references to the model (34). Tuckman proposed four stages of group development: (1) Forming, (2) Storming, (3) Norming, and (4) Performing. This model, introduced in 1965, has stood up over the years and continues to be the predominant theory of group development.

Several common challenges faced by interdisciplinary teams have been identified. Some of these include toxic leadership (35, 36), large team size, goal misalignment between the investigator’s needs and team’s goals, lack of common scientific vocabulary, institutional disincentives and competing priorities regarding career advancement (25, 37). However, the overwhelming barrier to robust collaboration at many institutions continues to be limited time and funding (38, 39). This is a brief list of the typical challenges faced by multidisciplinary teams, and more on this can be found in the literature. In the sections that follow, we elaborate on several of these challenges that impacted the COVID-19 Research Initiative.

### 2.5 Virtual team infrastructure and team initiation activities

After the investigators chose teams from the list provided in the REDCap survey, the CTSI staff worked around-the-clock on April 8^th^ and 9^th^, 2020, to create each team’s Microsoft Teams workspaces populated with team members. Each of the 15 project managers immediately emailed their team members on April 9^th^ to introduce themselves and schedule team meetings, which for some, began on April 10^th^. Each project manager was assigned 1–4 research teams.

### 2.6 First goal of team implementation: team charter development

By May 8^th^, 2020, each team had created a Team Charter that provided the team’s Research Question, Research Category, Team Membership, and Research Purpose Statement. The charter also contained Team Leadership, Potential Team Products, Specific Aims, RFA Targets, Research Expertise Gaps, and Access Needed to Specific Resources (e.g., COVID-19 mice, COVID-19 virus, or Biosafety Level 3 access). The Team Charter was used by CTSI leadership to gauge the progress and needs of teams. As the initiative gained momentum, teams continued to update their Team Charters with ongoing developments (See S5 for Team Charter example).

### 2.7 Simultaneous building and implementation of the COVID-19 research initiative

Although significant work occurred during the two-week planning phase for the rollout and implementation of the COVID-19 Research Initiative, most of the infrastructure for this initiative was designed and built simultaneously “on the fly,” while the initiative was in full progress. During the first 6 weeks of the initiative, this required a daily regimen of designing, building, and deploying research infrastructure, and nightly evaluation of each day’s progress and development of next-day activities. Below are two key areas of infrastructure that were built with expedience in the early weeks of the initiative.

#### 2.7.1 REDCap development for organization and progress tracking

REDCap forms were created to capture (1) team membership, (2) demographics of team members, (3) progress towards developing team scope, and (4) team meeting dates and activities. At a later date, a fifth form was developed to track team projects and metrics for productivity, including specific aims written; literature search conducted; RFA identified; grant submitted/status; regulatory approvals; manuscript submissions, publications and presentations; and CTSI services used. Components of the REDCap tracking system were, in some cases, created after some team activities had already taken place, and in these cases, project managers entered data retrospectively. CTSI services and supports were tracked using both the REDCap database and other tracking mechanisms, including Microsoft Outlook meeting records and a master log of all administrative meetings.

#### 2.7.2 COVID-19 research initiative webpage and COVID-19 research index

A webpage was created prior to the town hall meetings to describe the team nucleation process and provide essential information and a link to the Investigator Roadmap PDF. Soon after, a Research Index Page was created where team information was displayed as it became available, including Team Name, Research Scope, Team Leaders, Team Members, and contact information for project managers. This index also listed COVID-19 clinical trials from other non-CTSI initiatives at F&MCW (index no longer available).

### 2.8 Project management training and priorities

For this initiative, most of the 15 CTSI staff and faculty, as well as 3 staff from the MCW Cardiovascular Center, were given new roles as COVID-19 project managers. Before the pandemic, these individuals held positions such as research coordinator, project coordinator, data operations manager, program director, project manager, administrative assistant, and CTSI faculty leadership. Several of the COVID-19 team project managers began with only a modest project management background, and little or no direct exposure to the Ensemble concept. To address this gap, CTSI quickly developed and implemented a project management/Ensemble/team science training class and provided weekly virtual “walk-in office hours” for training and troubleshooting project management challenges. CTSI also held training sessions on grant components and fundamentals, institutional grant submission processes, and the basics of interpreting an RFA.

### 2.9 CTSI administrative, technical, and research support for team implementation and productivity

CTSI faculty and staff provided all administrative support for the COVID-19 Research Initiative. Additional CTSI supports were made available as needed, including services for (1) Creating an Operational Plan, (2) Clinical Trial Protocol Development, (3) Clinical Trial Execution, (4) Biomedical Informatics, (5) Community Engagement and Research, and (6) Biostatistics. CTSI also created a comprehensive process to curate new COVID-19 funding opportunities and provide teams with daily alerts for new RFA opportunities.

## 3 Results

### 3.1 Naming teams: unique COVID-19 C&TR questions provide initial scope of teams

A comprehensive search of COVID-19 grant resources identified 50 unique extramural federal and private funding opportunities for COVID-19 C&TR. Additional C&TR questions were provided by the Leadership Advisory Committee (20 questions) and research faculty at UWM (47 questions). A total of 117 potential C&TR questions were reviewed to eliminate redundancies, combine similar questions, and remove non-translational goals. The 31 remaining C&TR questions were used to nucleate investigators into 31 initial research teams. These research teams were grouped into five categories: (1) COVID-19 Treatment & Vaccine, (2) Health System Reform, (3) Clinic Environment, (4) Epidemiology, and (5) Communities (Table 1).

**Table 1:** Investigators formed 31 initial teams by nucleating around unique COVID-19 C&TR questions.

COVID-19 Treatment and Vaccine Develop new therapeutics that can be administered in a non-hospital-based setting in the management of COVID-19. Short-term project (9-12mo) focusing on COVID-19 and Cardiovascular System. Explore technologies that are in final stages of R&D to manage COVID-19. Develop unique model systems to explore current and inform future prevention, diagnosis, and treatment of HLB conditions affected by Coronavirus. Create a team science-based solution to management of COVID-19 that will be broadly applicable at all CTSA sites. Collect feasibility data for conduct of future innovative Phase ½ clinical trials using existing drugs and biologics. What medications are contraindicated in COVID-19 therapy? What mechanisms can be harnessed to develop COVID-19 antiviral therapy? Health System Reform What changes will COVID-19 bring to clinic workflow in a post-pandemic environment? What shovel-ready healthcare reforms can address overwhelming large-scale health crises? Projects to address timely health system and healthcare professional response to COVID-19 and like crisis. Management of national-level emergencies through modernization and optimization of existing hospital systems, governance structures and informatics tools. What is effective education and dissemination of information in a crisis scenario? Clinic Environment Can we improve COVID-19 PPE effectiveness, sterilization, and durability? Among emergency medicine staff, which if any workers develop COVID-19 immunity? How can we maintain effectiveness of COVID-19 emergency medicine environments? Registry to develop the infrastructure necessary to identify healthcare workers at high risk for COVID-19 who may be eligible for participation in future clinical trials of COVID-19 prophylaxis and treatment. Develop novel training and education modalities to reduce exposure risk to health workers. What is psychological stress from COVID-19 on healthcare workers? What is the impact of the pandemic on the management and allocation of OR resources? Epidemiology Develop COVID-19 Diagnostic Tools. How to increase COVID-19 testing capacity? Generate timely evidence on the direction and magnitude of association between the use of ACEI or ARBs and COVID-19 severity and mortality. Develop Point of Care testing for COVID-19 and other infectious diseases. Genomic studies to explore why children are not as affected. Study life history of COVID-19 to develop better predictive models of spread, transmissibility and interventions. Use of biomedical informatics tools to better understand and manage COVID-19. What are the psychological sequelae from COVID-19? Communities What are COVID-19 complications in high-risk communities? Study of risks and outcomes of COVID in substance abusers. Genetic susceptibility to COVID-19 across race.

### 3.2 Team formation results and early implementation assignments

**Figure 2:**
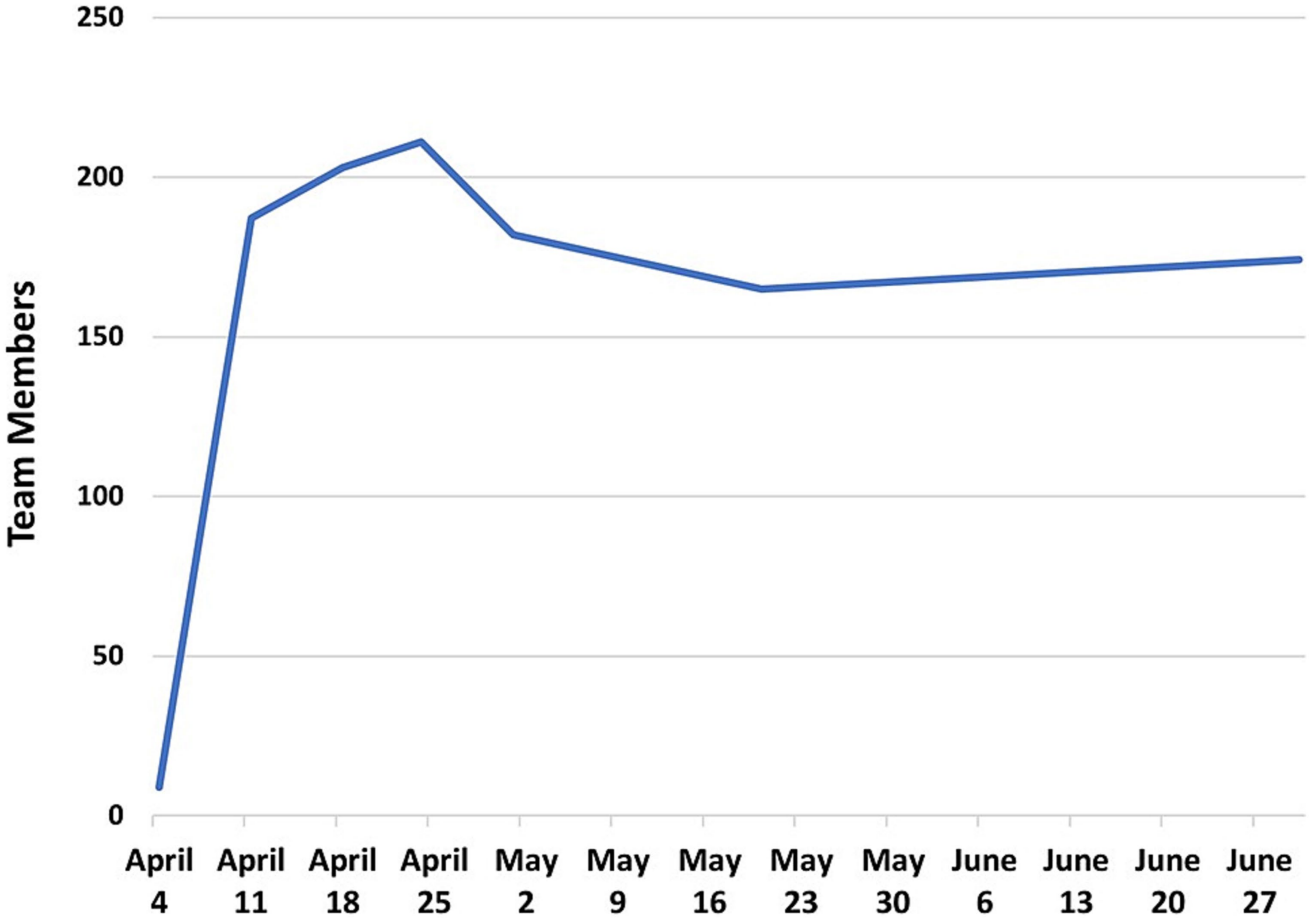
Total investigators added to COVID-19 teams through April 24^th^, 2020.

After the April 8^th^ Town Hall Meetings, the Call to Action immediately attracted 175 investigators within days, with a peak of 211 investigators by April 24^th^, 2020 (Figure 2). Of the 205 investigators who joined the initiative, 120 were from MCW and 85 were from CTSI partner institutions.

**Figure 3:**
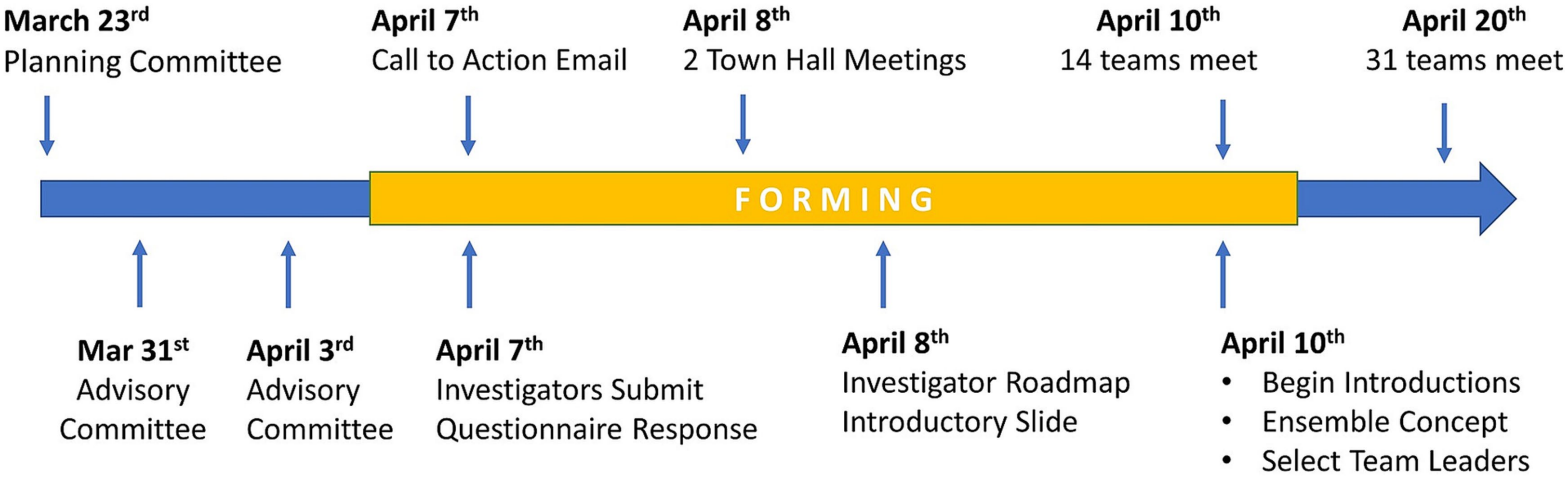
Early milestones of the COVID-19 Research Initiative.

On April 10^th^, 2020, 14 teams held their first meetings using the Microsoft Teams virtual meeting platform. By April 20^th^, 2020, 30 teams had met at least once. At this point, the teams ranged in size from 3 to 35 members. The early milestones of the COVID-19 Research Initiative are shown in Figure 3. During this early phase of the initiative, teams were given assignments to (1) finalize team leadership, (2) formulate their research scope and specific aims, (3) identify RFA targets, and (4) create a team charter. These goals were part of the general implementation strategy for all teams. However, individual teams developed at their own pace, and may have used different implementation strategies. Unfortunately, documentation of individual team implementation steps was not possible, since project managers were fully occupied by priorities to accelerate team objectives and document metrics of productivity.

### 3.3 Team member profiles: faculty rank and institution, team member role, research experience

**Figure 4:**
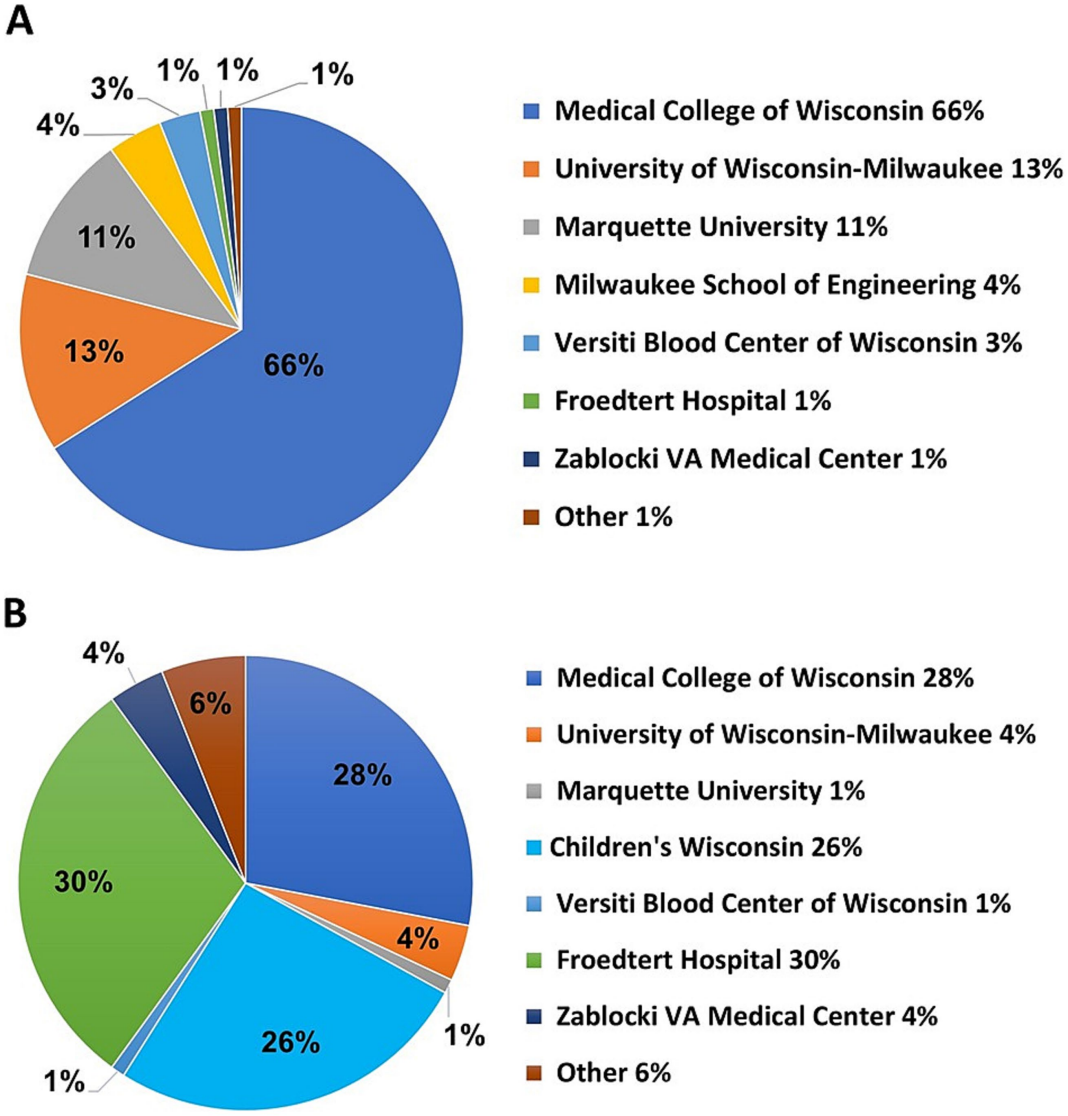
**(A)** Team member primary institutions obtained from a survey of 174 investigators. **(B)** Team member secondary institutions.

From April 10^th^, 2020, to April 30^th^, 2021, a total of 243 team members participated in COVID-19 research teams for some duration. On June 25^th^, 2020, active team members (*n* = 174) were asked to respond to a REDCap survey designed to obtain additional demographic information. Responses from 119 (68%) survey participants indicated that team members were predominantly from MCW (*n* = 79, 66%), followed by UWM (*n* = 15, 13%), and Marquette University (*n* = 13, 11%) (Figure 4A). Among team members that indicated a secondary institution (*n* = 78), Froedtert Hospital (*n* = 23, 30%) ranked the highest, followed by MCW (*n* = 22, 28%) and Children’s Wisconsin (*n* = 20, 26%) (Figure 4B).

**Figure 5:**
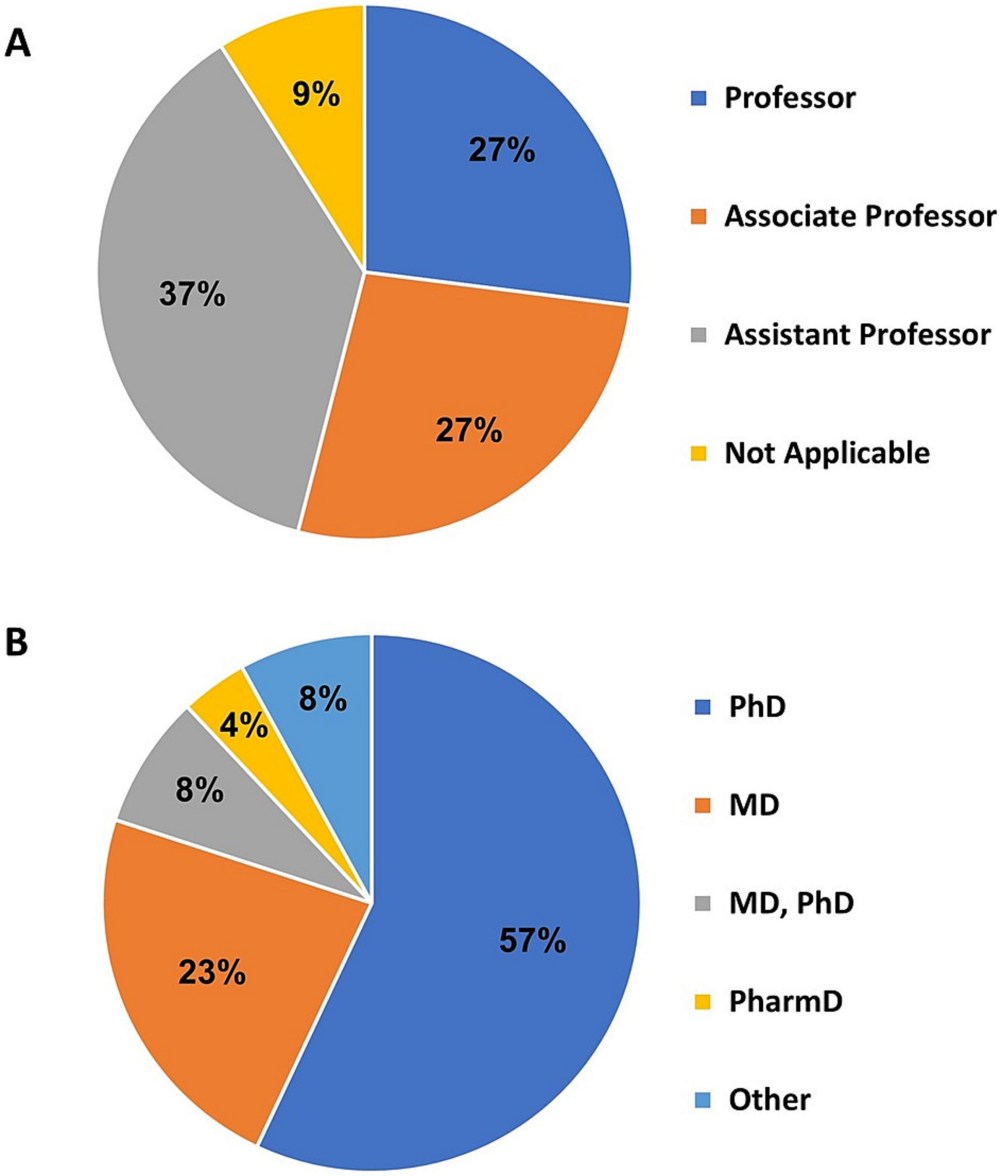
**(A)** The academic rank of investigators obtained from 116 survey responses. **(B)** Reported degrees of investigators. “Other” indicates one of the following: DO, PsyD, DNP, MS, MBA, MPA, BSN, BS, RN license.

As shown in Figure 5A, among the 116 responses, the most prevalent faculty rank was assistant professor (*n* = 44, 37%), followed by associate professor (*n* = 31, 27%) and professor (*n* = 31, 27%), and non-academic (*n* = 10, 9%). Among 119 respondents, the most common degree was PhD (*n* = 69, 58%), followed by MD (*n* = 27, 23%), MD & PhD (*n* = 9, 8%), and PharmD (*n* = 5, 4%). There were 10 (8%) responses that indicated one of the following degrees: DO, PsyD, DNP, MS, MBA, MPA, BSN, BS, (Figure 5B).

**Figure 6:**
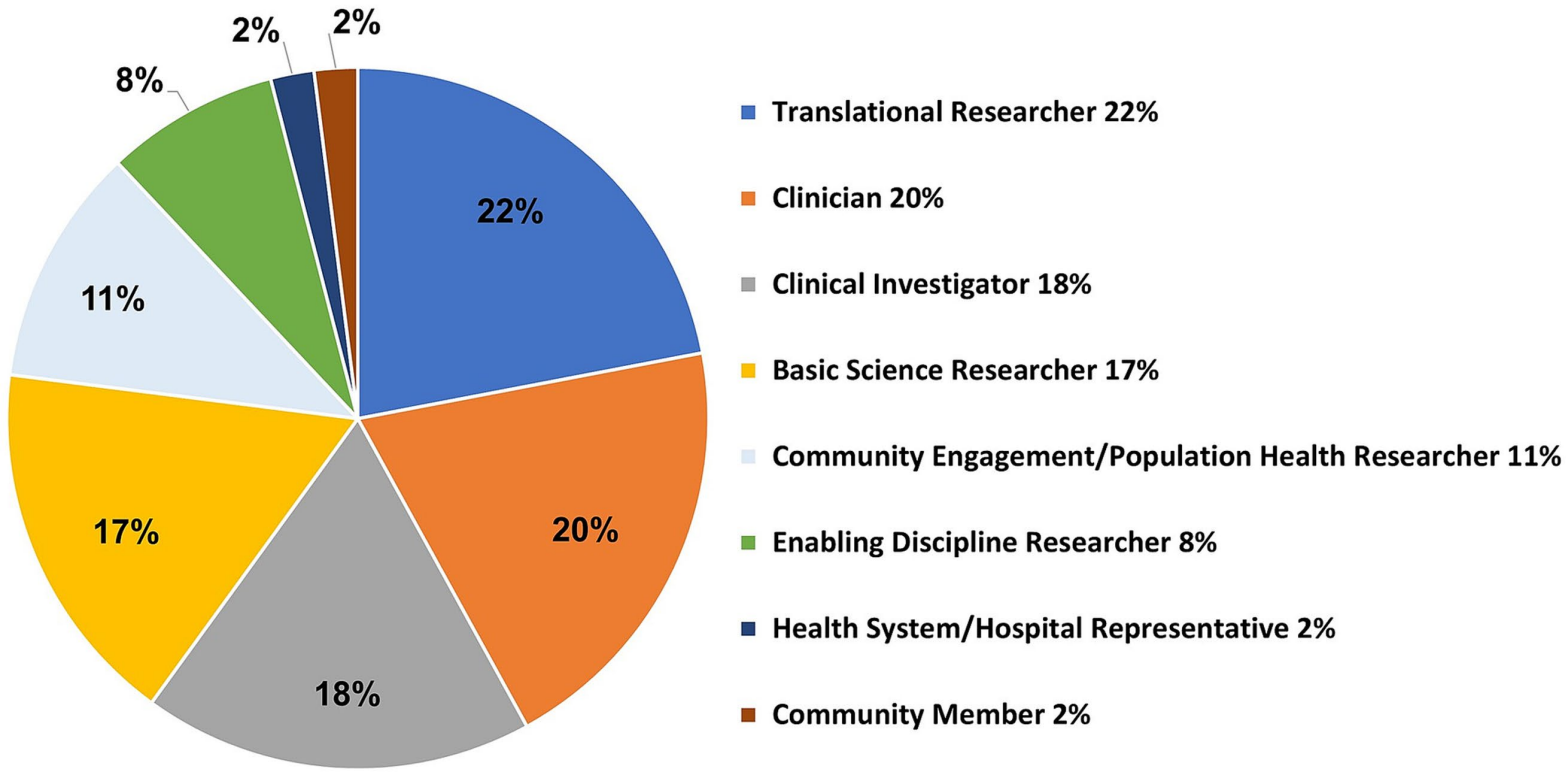
CTSI Ensemble-defined investigator team roles obtained from 118 survey responses.

The Ensemble concept leverages multidisciplinary team member participation, and the type of participation is characterized by Ensemble ‘roles’ as previously discussed (Section 2.4.2; Figure 1B). Team members were asked to report their Ensemble role in the COVID-19 Research Initiative. Survey respondents could select one or more team member roles, and examples were provided for some choices. Survey respondents (*n* = 118) listed a total of 202 roles, with the most predominant roles being Translational Researcher (*n* = 51, 22%) and Clinician (e.g., MD, physician assistant, nurse, etc.; *n* = 47, 20%), followed by Clinical Investigator (*n* = 41, 18%), Basic Science Researcher (*n* = 40, 17%), Community Engagement or Population Health Researcher (*n* = 25, 11%), Enabling Discipline Researcher (e.g., Biostatistician, Data Scientist, Bioinformatics, Epidemiologist, Genomics Support, etc.; *n* = 20, 8%), and Health System or Hospital Representative (e.g., Division Administrator, Clinic Manager, etc.; *n* = 5, 2%), and Community Member (*n* = 4, 2%)(Figure 6).

**Figure 7:**
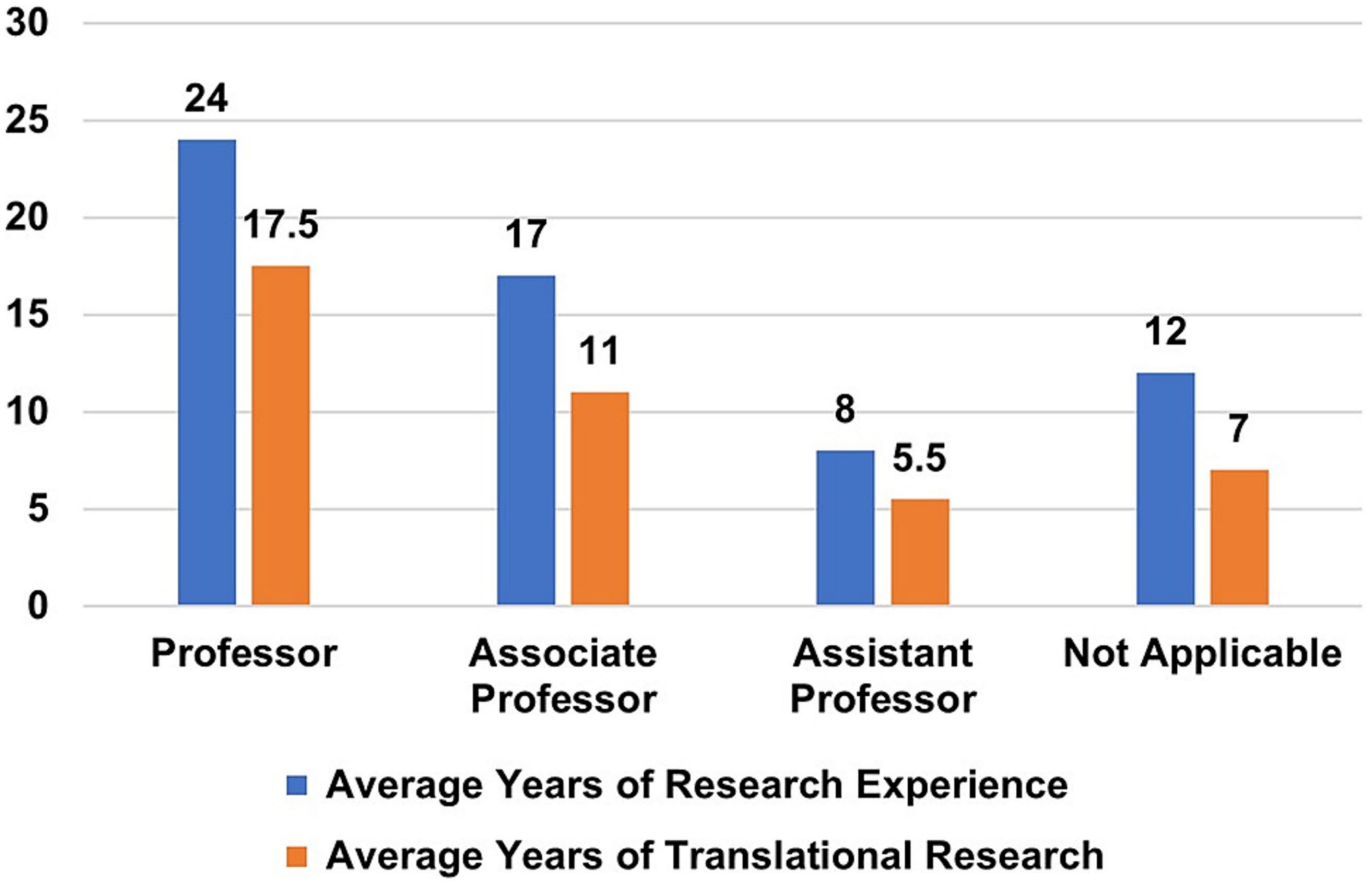
Years of research experience vs. translational research experience according to academic rank.

Among team members who responded (*n* = 119), the number of years’ experience in translational research and overall research ranged from 5 to 17 years (average = 10.38 yrs) and 8–24 years (average = 14.97 yrs), respectively. Professors (*n* = 31) reported the greatest amount of translational experience (average = 17.5 years), and Assistant Professors (*n* = 31) reported the least amount of translational experience (average = 5.5 years) (Figure 7). Six team members reported no previous experience in research, and 16 investigators reported no previous experience in translational research. At the time of the survey, the demographic profiles of active team members and withdrawn team members were comparable (data not shown).

### 3.4 Progress reporting

From April 10^th^ to May 1^st^, 2020, the Planning Committee and Project Manager Group held twice-daily meetings to discuss team progress, challenges, and potential solutions, and assign resources to address barriers. At each meeting, 2–3 project managers were scheduled in rotation to provide progress updates. Beginning in May 2020, the frequency of meetings was reduced to once a day, and from August 2020 to February 2021, the frequency was gradually rolled back. By March 2021, these meetings were held monthly, and in January 2022, these meetings were phased out. Progress of the overall initiative was shared at (1) a meeting of all COVID-19 team members on May 20^th^, with a total of 93 team members in attendance, and (2) a meeting with all CTSI staff and faculty on July 20^th^, 2020.

### 3.5 Faculty and staff effort: planning, development, and execution of the research initiative

Although research teams received no institutional funding from F&MCW or any CTSI partner institutions, substantial faculty and staff support was provided to plan and administer the COVID-19 Research Initiative. Data on the number of meetings and time to prepare for the execution of the project was tracked for the period of March 23, 2020, to December 21, 2020, using the REDCap database, daily activity logs, and Microsoft Outlook calendars (S6). There was a total of 54 meetings (289 person hours) prior to the public rollout of the initiative, which included 11 meetings (221 person hours) for the planning and preparation phase and 34 meetings (68 person hours) for project management training and support. During the first year of the initiative, project managers provided support (278 person hours) for a total of 370 team meetings. Project managers also attended 135 CTSI administrative meetings (1,337 person hours). However, data was not tracked for creating the REDCap surveys and database, Microsoft Teams virtual spaces, Power BI reports, or webpages. Other administrative activities included daily searches for new COVID-19 funding opportunities, and data entry by project managers. By far, the largest investment of time came from the COVID-19 team members themselves, who devoted substantial time to refine research ideas, submit grant proposals, conduct research projects, and present and publish research findings.

### 3.6 Team science challenges

#### 3.6.1 Scope and size of teams

After the initial nucleation step, some of the 31 teams began to subdivide into two or more smaller teams. In some cases, the team’s scope was too broad and/or the teams had too many members to be practical. Some teams also merged because their research questions were similar or related. This brought the total number of teams formed over the course of the initiative to 41. In addition, seven teams modified their team names to reflect the evolving scope of their research.

#### 3.6.2 Team science challenges cause members to withdraw from COVID-19 research teams

**Figure 8:**
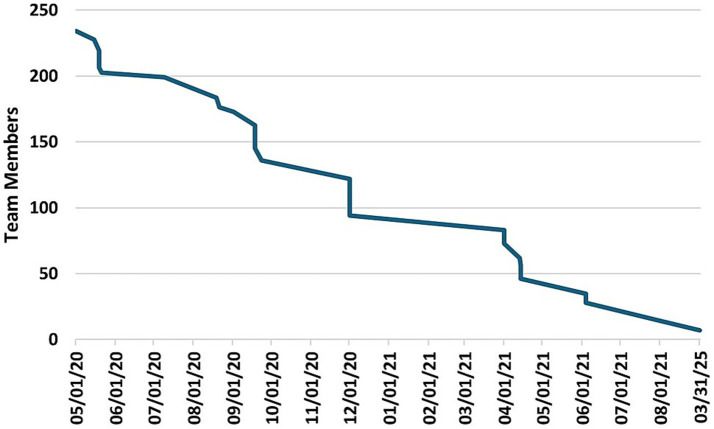
Total number of investigators participating in the initiative from May 1, 2020 to March 3, 2025.

After an initial peak of 212 team members on April 25^th^, 2020, several investigators gradually began limiting their participation across teams, migrating to different teams, or withdrawing from teams. By June 18^th^, 2020, 53 investigators had left the initiative, prompting CTSI to examine the reasons for their departure. These investigators were emailed a request to provide reasons for leaving the initiative and to rate the reasons according to importance (1–10), where 10 was most important. Twenty-two participants responded with 51 reasons for leaving the COVID-19 Research Initiative. The reported causes were grouped by similarity into six categories that reflected either “Personal Issues” or “Team Issues.” Table 2 provides representative examples of the responses, and the number of times reported. The highest frequency of responses concerning Personal Issues and Team Issues, respectively, were Bandwidth or Work Time Constraints (17 mentions; 33% of all responses) and Poor Team Communication or Team Structure (8 mentions; 16% of all responses). The average rank of importance for the responses was higher for Personal Issues (8.7) versus Team Issues (7.4). Figure 8 illustrates the investigator attrition curve over time.

**Table 2:** Challenges to participation provided by team members departing the COVID-19 Research Initiative.

Personal Issues or Team Issues	Themes based on issues that prevented participation, and relevant examples
	Bandwidth/Work time constraints (Reported 17 times)
PERSONAL ISSUES	Increased workload and stress associated with work-from-home orders, and it was difficult to prioritize. I had to ultimately focus on imminent grant submissions, finishing up teaching this semester, and submitting a manuscript. I could not participate as I have a huge amount of clinical work plus all the research we are performing. I have had to cover extra calls too. I had too many conflicting meetings. I ended up on a couple of COVID-19 surge planning committees. An Insufficient number of hours in the day. Time—this was a time of great transition for our school, as with all higher learning institutions. As the Dean of the School of Nursing, I had many commitments and meetings to keep courses running with my faculty, to keep satisfaction with students, and to complete future planning with leadership. Personal lack of time
	Lack of expertise or interest in team topics/focus (Reported 9 times)
	None of the initiatives were really relevant to my area of research. I attended the kick-off meeting and found the team discussion was a little far from my research expertise. The proposed projects did not match my primary populations or social problems of interest. There wasn’t enough overlap between my area of expertise and the research directions that were being discussed at the meetings to warrant spending significant time on these research efforts. The group that I got into did not fit my interests
	Other grant/research project commitments/career priorities (Reported 8 times)
	Being on tenure track, I want to ensure that I do not overextend myself and take time away from producing first authored manuscripts and securing external funding. Adding additional meetings and projects would take away from this time. I participated in several BMT-specific COVID-19-related research initiatives that took a lot of my time; there did not seem to be an opportunity or sufficient time to integrate that into CTSI initiatives. Ended up receiving a large grant that has occupied all of my time. Involved in the submission of three COVID-19-related grants.
	Family/Personal responsibilities (Reported 2 times)
	I have three kids aged five and under. Work/home balance was difficult and meant that I had to prioritize and focus on more urgent/required needs. I have less time to devote to other things. Family obligations; Initially I was working from home and I was able to do a majority of the childcare for our three children. With that in mind, I believed that I would have more time to involve myself in other projects. Within a week of signing up, my wife was required to begin teaching her classes online; I had to focus on my primary workload from Children’s and assist her with childcare.
TEAM ISSUES	Poor team communication/team structure (Reported 8 times)
	Did not like the group structure-another meeting to set up a meeting etc. Seemed like a waste of time. I found the process overall to be a bit overwhelming. I ended up being on multiple teams, it was unclear what the necessary tasks were (e.g., presentation of ideas, etc.) for each. Difficult to know where a project “fit.” I did not understand there would be so many teams. I checked the boxes that I had expertise/interest in. Then it turned out to be multiple teams and meetings, some even at the same time. Obviously, I did not have time for all of them.
	Lack of team direction/leadership/funding (Reported 7 times)
	The group that I ended up in was a group of providers whom I did not have a shared sense of direction with. Lack of clarity on the process of the research initiative and end goal/outcome (despite attending the intro meeting) Unclear direction/leadership/structure It appears 1 or 2 projects would come from the teams I was a part of, but it did not look as though those would be fundable. As mine is a grant-funded position, I really need to be involved in things that will bring in funding. Lack of funding The group in which I was interested dissolved.

#### 3.6.3 Evolving pandemic priorities cause some teams to retire

**Figure 9:**
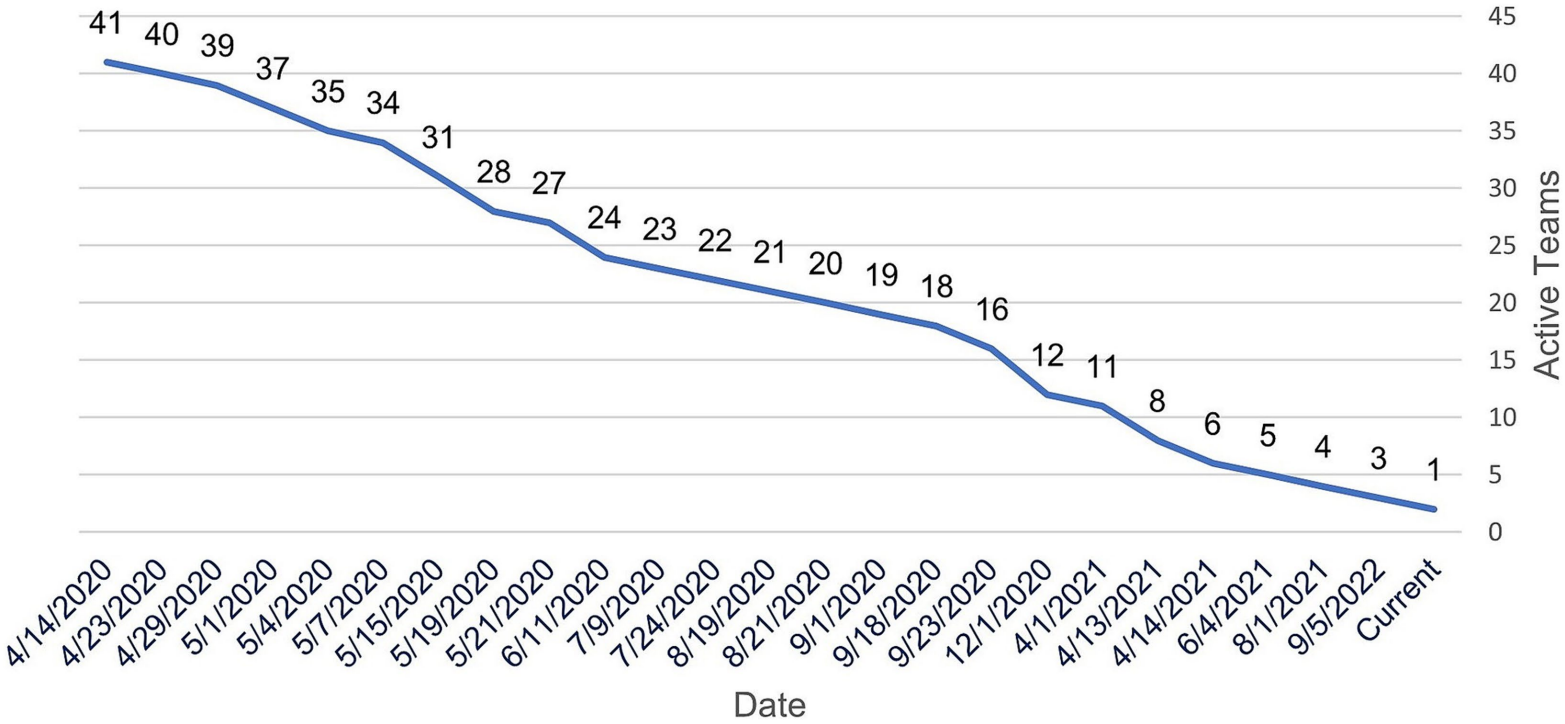
Teams remaining active vs. time.

Coinciding with team member withdrawals, COVID-19 teams also began to retire as shown in Figure 9. These teams ended primarily as a result of departing team members that faced pivotal team science challenges, e.g., lack of funding opportunities to support research-track faculty, scheduling conflicts among members, clinical duties, investigator bandwidth, and goal misalignment of investigators. However, teams also retired for reasons that were driven by the evolving pandemic. For example, the funding landscape was quickly changing in response to new COVID-19 data, and research priorities at the national level were sometimes confusing or uncoordinated. A few teams with a narrow research focus found that their research question had become less imperative, or even obsolete.

For struggling teams, project managers worked with team leaders and their members to overcome obstacles, help recruit additional team members, or provide suggestions to revive teams after initial participation and enthusiasm had waned. Project managers and team members jointly made determinations to retire a team after considerable dialogue and failed attempts to reverse the team course. Ten teams retired in the first month of the initiative, and after the first year of the initiative, just eight teams remained active. As of the submission date of this manuscript, one team remains active.

### 3.7 Number of meetings per team

**Figure 10:**
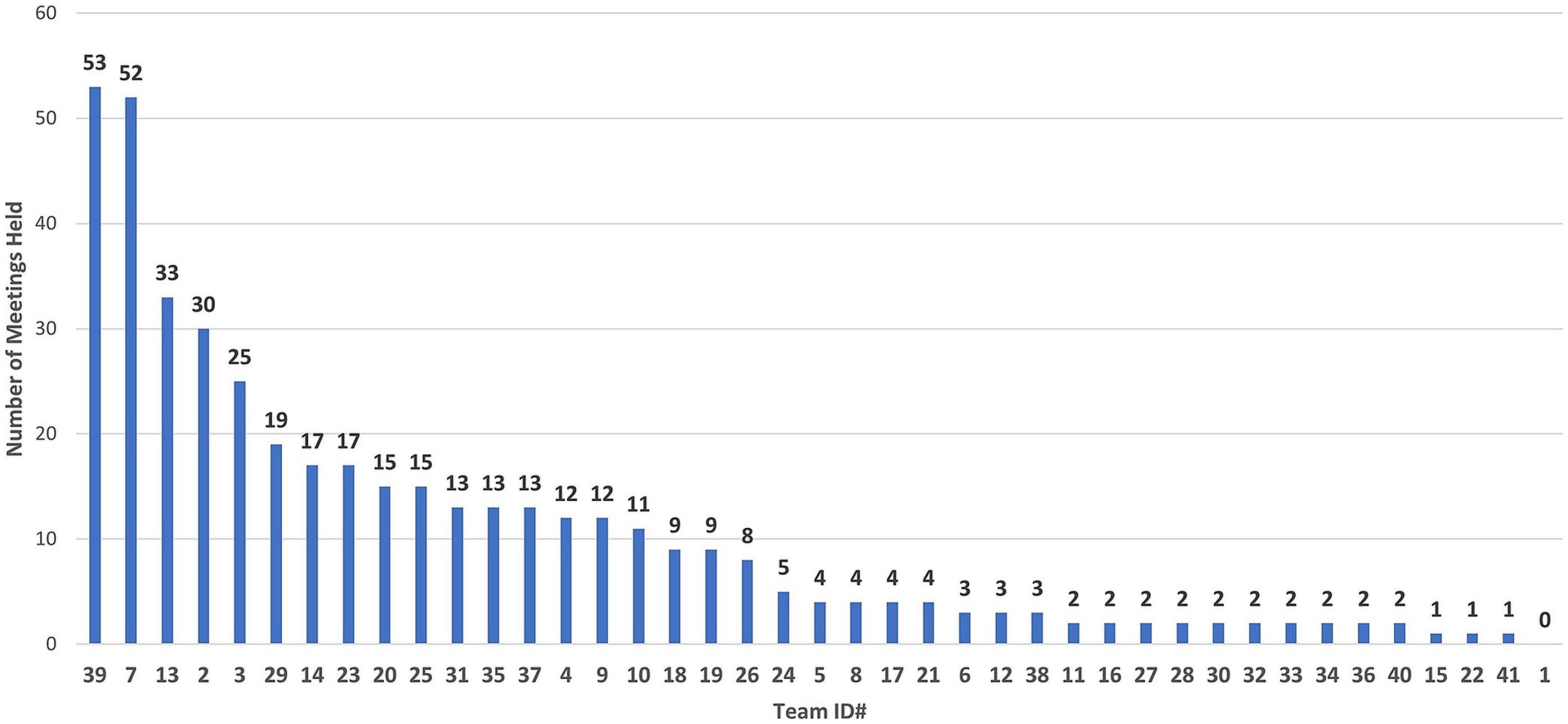
Total meetings per COVID-19 research team, from April 10, 2020, to December 21, 2020. Team #1 formed, but did not participate in the COVID-19 Research Initiative.

An analysis of the total number (*n* = 370) of meetings for all COVID-19 teams through December 21, 2020, found that (1) active COVID-19 teams had an average of 19 meetings, (2) retired COVID-19 teams had an average of 5 meetings, and (3) merged, subdivided, and dormant teams had an average of 2, 2, and 8 meetings, respectively (S7). For this analysis, the data cutoff was December 21, 2020, because afterward, data entry became sparse as a result of project managers adding new CTSI priorities. The total meetings held for each of the 41 teams are shown in Figure 10.

### 3.8 Team effectiveness metrics

The COVID-19 Research Initiative was collectively responsible for the submission of 20 grant proposals, totaling $32,528,297 ($25,482,280 in direct costs, and $7,046,017 in indirect costs). Three grant proposals were funded, totaling $539,790 in direct costs and $70,098 in indirect costs, while 17 grant proposals were declined. There were four manuscripts published during the initiative (40–43), four manuscripts published afterward (44–47), and eight presentations (48–55). Other productivity included: (1) approval for IRB protocols (3 teams); clinical protocols for grant submissions that were declined (2 teams); and (3) a survey with 322 respondents (1 team). Teams used The following CTSI research services: Project Management (41 teams), Biostatistics (5 teams), Community Engagement and Integrating Special Populations (3 teams), Clinical Trials Office (1 team), Translational Research Unit (1 team), and utilization of Trial Innovation Network (TIN) for protocol development, statistics and budget development (1 team; TIN is an initiative of the CTSA for coordinating C&TR over multiple institutions) (56). In total, 12 COVID-19 teams had scholarly output that included either grants (submitted or awarded), publications, or presentations. These results are summarized in S8.

### 3.9 Case studies of teams with significant impact

#### 3.9.1 Example of multidisciplinary team expertise partnering with community stakeholders

The early work of the *Community Responsive Communications* team led to a shared vision that focused on a local target of their research:

*“The absence of reliable COVID-19 health information in Milwaukee African American and Hispanic communities, combined with a lack of trust in the medical establishment, is a significant barrier to health care access.”*

To better understand this barrier and identify the community’s needs during the pandemic, the team recruited representatives from the (1) African American Leadership Alliance Milwaukee, (2) Hispanic Collaborative of Milwaukee, and (3) Sojourner Family Peace Center (Milwaukee). The inclusion of these community stakeholders helped narrow the scope to intimate partner violence (IPV), which had increased as a result of social isolation and financial stress during the pandemic. Members of the team created a shared vision and project goals, which included (1) the placement of trained communication advocates for domestic violence screening in hospital clinic waiting areas, and (2) the implementation of a screening survey tool to identify individuals at risk of, or experiencing domestic violence, and refer those participants to resources at the Sojourner Family Peace Center. The team consisted of 15 multidisciplinary members with interinstitutional integration and representation from each of the three domains of the Tri-lateral Mutually Learning Ecosystem: (1) Clinical Translational Research Enterprise, (2) Health Care Systems, and (3) Community of Stakeholders (Table 3).

**Table 3:** Ensemble teams are intentionally multidisciplinary in composition.

Team Member Expertise Applied Gerontology and Social Work Clinical Laboratory Science Community Engagement Diversity and Inclusion Initiatives Domestic Violence Intimate Partner Violence Obstetrics & Gynecology Nursing Research Methods, Outcomes, and Evaluation Pediatric Gastroenterology & Nutrition Pediatric Psychiatry Women’s Health
Team Member Ensemble Roles (number of members) Domain: C&TR Enterprise Basic Science Researcher (1) Clinician (1) Clinical Investigator (4) Translational Researcher (1) Domain: Community of Stakeholders Community Member/Patient (2) Community/Population Health Researcher (3) Enabling Discipline Researcher (1) Domain: Health Care Systems Health System/Hospital Representative (2)
Team Member Institutions African American Leadership Alliance, Milwaukee, WI Ascension St. Mary’s, Milwaukee, WI Children’s Hospital of Richmond at Virginia Commonwealth University, Richmond, VA F&MCW Hispanic Collaborative of Milwaukee Marquette University, Milwaukee, WI Milwaukee School of Engineering, Milwaukee, WI Sojourner Family Peace Center, Milwaukee, WI University of Wisconsin—Milwaukee

The Community Responsive Communications team has a wide range of expertise and Ensemble roles, as defined by the Ensemble model of team formation (Section 2.4.2; Figure 1). This team also has significant multi-institutional team membership and partnerships with community organizations.

Very importantly, two team members had experienced intimate partner violence firsthand. Their input was vital to the development of the team’s goals and implementation of a tool to screen for intimate partner violence. They helped devise strategies for approaching people in a discreet manner that respected privacy, made people feel comfortable, and maintained caution to prevent alerting potentially violent partners. Other members of the multidisciplinary team included “Health System/Hospital Representatives” who provided valuable insights to implement the intervention in clinics at F&MCW.

In June 2021, the team secured a 3-year, $384,979 award, *“Integrating advocates within a healthcare setting to strengthen intimate partner violence screening (PI, Kimberly Gecsi, MD),”* funded by the Advancing a Healthier Wisconsin Endowment (AHW). As part of the research study, The team implemented a new procedure to screen patients for IPV at the F&MCW Obstetrics and Gynecology Clinic. The study had several important impacts: (1) 355 patients were screened by providers and advocates who underwent intensive training with the IPV screening tool; (2) Trained advocates and providers identified 37 patients who were referred to a social worker; (3) There was an increased rate of identification using the new procedure, when compared to historical data; and (4) Clinic procedures changed from screening patients by phone, to screening patients in person. The AHW grant resulted in two additional publications (46, 47). The *Community Responsive Communications* team is the only remaining active team from the initiative.

#### 3.9.2 Impact of two teams on hospitalized COVID-19 patients and frontline workers

The work of two COVID-19 teams had an immediate impact on both patients and hospital staff at F&MCW. The first team, *What is Psychological Stress from COVID-19 on Healthcare Workers?,* obtained a $25,000 grant (PI, Kristin Kroll, PhD) from the Greater Foundation of Milwaukee to create a virtual emotional support clinic for hospital staff, called the *BRaVe Clinic: Building Resilience Virtually.* The team consisted of psychologists, pharmacists, and physicians from different fields, as well as experts in nursing and social work. The clinic was entirely virtual and provided training for doctoral students on delivering evidence-based care via telebehavioral health. During the COVID-19 Research Initiative, the BRaVe Clinic treated 62 clients from June 19, 2020, to September 30, 2021 (Personal communication from team member, Courtney O. Barry, PsyD; F&MCW). At the time, this was a truly unique opportunity for students, since few student training opportunities existed in telebehavioral health. As part of this team’s accomplishments, their work resulted in the development of a virtual consent process used in other clinics, and led to additional behavioral health interventions, not only for support of F&MCW faculty, staff, and students, but also for support of Milwaukee residents, regardless of insurance status (43).

The second COVID-19 team was named *Percutaneous Right Ventricular Assist Device (RVAD) for COVID-19 Acute Respiratory Distress Syndrome (ARDS)*. This team studied the novel use of extracorporeal membrane oxygenation (ECMO) with RVAD in patients with severe COVID-19 ARDS. The team’s clinical trial of 39 patients with ARDS demonstrated that RVAD support at the time of ECMO initiation resulted in higher in-hospital and 30-day survival, and no secondary end-organ damage, when compared to invasive mechanical ventilation, alone. This team’s research resulted in an oral presentation at the Academic Surgical Congress (55) and was published afterward (40). Further research led to another publication (44) by the team’s leader, Michael Cain, MD, and drove greater interest in right ventricular protection in ARDS. These research findings were recently published within international guidelines (57).

## 4 Specific team challenges, lessons learned, and suggestions to improve future research initiatives

CTSI created and executed the COVID-19 Research Initiative with remarkable speed and efficiency that generated an unprecedented acceleration of multidisciplinary team formation from investigators throughout Southeast Wisconsin. There were other initiatives at CTSI, and in fact, CTSI was funded with an NCATS supplemental grant (“RADx Underserved Populations”) to (1) determine the COVID-19 prevalence in Milwaukee County, (2) understand the risks of infection, and (3) inform decisions to reduce personal risk (10, 11, 23). In addition, CTSI obtained NIH funding (3UL1TR001436-06S2) and partnered with Advocate Aurora for the clinical trial, “CONTAIN COVID-19: Convalescent Plasma to Limit COVID-19 Complications in Hospitalized Patients” (5). These studies were funded through the NIH and yielded significant results with local and national impact. In comparison, teams of the COVID-19 Research Initiative received no funding and were expected to secure their own individual grant awards and resources. Hence, the expectations and experiences of the COVID-19 Research Initiative need to be viewed through this important lens. Overall, this experience provided significant insights and lessons, and below we present these for informing the design of future accelerated research initiatives.

### 4.1 Early team science challenges

#### 4.1.1 Difficulties with technology for virtual collaboration

Prior to the COVID-19 pandemic, the challenges to team science from long-distance collaboration and virtual communication were already known (27, 58). Since then, the impact of virtual communication in the workplace has continued to be studied and debated (59–61). Our research initiative brought together investigators from Southeast Wisconsin academic institutions who were no more than 15 miles apart. Despite the short distance, the complete shift to virtual communication presented technology and communication challenges akin to long-distance collaborations. For example, there were firewall security-related issues, experienced by team members at non-F&MCW partner institutions, which delayed the addition of investigators to their virtual teams. These delays, lasting 3–4 days, were also fueled by the overwhelming demand for remote online work and reduced capacity of Microsoft servers in the first weeks of April 2020 (62). The online delays added to a sometimes confusing process, but fortunately, this was a short-term problem. Other issues included difficulties with basic navigation of the virtual workspace: What seems today like an easy technology to master, was at the time, a significant, and occasionally steep learning curve for some team members. In addition, team members from institutions external to F&MCW could not access real-time modifications to documents stored on Microsoft Teams. These access issues became challenging when collaboration was required to develop or edit a single document, making work-arounds a necessity. The virtual environment also affected the early planning activities by CTSI leadership, faculty, and staff, who sometimes faced significant difficulties with the smallest of virtual collaboration challenges. At times, planning returned to known technologies, like using a cell phone for conference calls. As a result, during some of the most important, early stages of planning, expedience predominated over the priority for more inclusive collaboration.

#### 4.1.2 Urgency prevents full collaboration during the planning stage of the COVID-19 research initiative

As noted above, the initial challenges of working remotely sometimes hindered broader input at critical junctures of planning the initiative. For example, the plans to nucleate investigators into teams might have benefited from wider collaboration. Only a few individuals from the Planning Committee came together to create this plan. In hindsight, the plan failed to address team membership limits and resulted in teams with too many members and investigators overextended by participation in too many teams. For some investigators, inefficiencies, and ambiguity in the nucleation process may have dampened their enthusiasm (e.g., ‘process-induced fatigue’) and, indeed, exit questionnaires indicated that some participants had experienced feelings of disorganization as teams began meeting (e.g., ‘debate-induced attrition of focus or scope’). Projects have an opportunity for greater success when a broad spectrum of stakeholders has the luxury of adequate time to collaborate (33). However, this “luxury” had to be weighed against other competing factors. While both the Planning and Leadership Advisory Committees provided significant multidisciplinary and multi-institutional efforts to the planning, the pace of planning activities and growing levels of urgency became a significant challenge to full collaboration during the planning stages. Moreover, some committee members felt we only had a small window of opportunity to capture the investigator’s attention and mobilize a significant research response. In hindsight, it seems reasonable to have extended the planning phase prior to the initiative kick-off.

#### 4.1.3 Underestimated volume and enthusiasm of participants causes formation of large teams and overcommitment of investigators

CTSI had no prior experience to anticipate the level of enthusiasm for the initiative, and therefore, did not hesitate to use a recruitment approach that maximized inclusion. There were no limits to the number of investigators who could sign up for a single team and no restrictions to the number of teams that investigators could join. In many cases, the lack of recruitment limits led to investigators signing up for multiple teams (sometimes, four or more teams), and led to the formation of some teams with as many as 35 apparent members. For some investigators, this immediately caused problems when they discovered that their teams were scheduled to meet at the same time. In addition, some investigators belonging to multiple teams became confused or overwhelmed by the large volume of team communications, numerous team activities, multiple MS Team meeting invitations, and the location of each team’s documentation. However, most team members were retained during this initial phase and continued collaboration.

By April 30^th^, 2020, we observed that many investigators were not participating in several of the teams that they had joined. Therefore, to maximize the effectiveness of these teams, CTSI contacted all investigators by email and requested that they evaluate their participation. Investigators were asked to assess their capacity to participate in multiple teams since overcommitment could become counterproductive due to limited investigator bandwidth, scheduling conflicts for teams that met concurrently, or investigator schedules that prevented sufficient participation in more than one team. CTSI recommended that investigators actively participate in only one team, and limit total participation to a maximum of three teams. In some cases, a team’s research question attracted exceptionally large numbers of investigators. This was the case with two teams: (1) “COVID-19 complications in high-risk communities” and (2) “Genetic susceptibility to COVID-19 across race.” The broad scope of these teams, combined with evidence-based concerns and investigator enthusiasm, led to a substantial number of investigators joining these two teams: 63 and 38 team members, respectively. It was immediately clear that the size of the two teams would cause difficulties, especially with forming a single shared vision and operating cohesively. This led to a decision to subdivide the team into smaller sub-teams, with a tighter research focus and fewer members. On April 27^th^, 2020, the project managers surveyed team members’ interests, resulting in the creation of four smaller teams: (1) Use of technology to address health disparities related to COVID-19; (2) HIV/AIDS and LGBTQA communities during and post COVID-19; (3) Health disparities: Health inequity challenges for African Americans during COVID-19; and (4) Genetic susceptibility to COVID-19 across race, ethnicity, and gender. With a smaller team membership and narrower scope, some of these newly subdivided teams had greater success that included improved scoping of team projects, creating team charters, and writing grant proposals.

### 4.2 CTSI project managers: stress, anxiety, workload, respite, wellness

Project management has been identified as a key component of team science for studies across the spectrum of C&TR (63). In the CTSI Ensemble Program, project managers are acknowledged as full members of research teams. This role comes with standard stressors, like any occupation, but the COVID-19 Research Initiative, with its accelerated pace and bold mandate, placed members of the CTSI project management team in multiple situations with elevated stressors. Project managers were immediately required to learn new roles, terminology, and technology systems. Some openly discussed these stressors during CTSI Planning Committee meetings or addressed them in private with their managers or colleagues. The new roles and sense of urgency placed some CTSI staff in uncomfortable dynamics during CTSI administrative team meetings. Many of the project managers were not accustomed to the occasional “clash of scientific ideas” that sometimes occurred under passionate debate. In response to growing concerns about potential stressors during the daily Planning Committee meetings, CTSI leadership worked with project managers and created a standardized agenda with guidelines on April 26^th^, 2020. The new guidelines and agenda added greater meeting structure, focus, and efficiency, which generated more constructive dialogue and collaborative discussion.

CTSI Project managers faced additional stressors outside of the workplace, brought on by the State of Wisconsin Department of Health Services Emergency Order #12 “Safer at Home” strict lockdown and quarantine measures (64). These stressors, documented by others, included home-schooling of children, potential job loss, social isolation, and of course, fears of acquiring COVID-19 infection (65). Some of these stressors were readily evident at MCW. For example, furloughs and salary reductions were implemented throughout MCW on April 21^st^, 2020, and CTSI temporarily lost some personnel. Fortunately, these financial mitigation measures were later reversed in July 2020, and no MCW personnel suffered a financial loss. Nonetheless, job insecurity undoubtedly remained a significant stressor that impacted a substantial portion of our research support personnel. This may not be surprising, since studies have documented the negative impact of job insecurity on mental health (65–67).

#### 4.2.1 CTSI provides mechanisms to support project managers

Early on, CTSI leadership recognized the signs of growing fatigue and stress among project managers and addressed these stressors in several ways. First, CTSI leadership was available and proactive in assisting individuals who reached out for emotional or technical help. In addition, CTSI team members were highly supportive of each other, and activities like group breathing exercises at the close of CTSI administrative meetings were also helpful.

Two members of the Planning Committee began a wellness initiative by creating a list of wellness exercises and making presentations to the CTSI administrative team and CTSI leadership. Project Managers could also take advantage of convenient one-hour “walk-in” training opportunities that allowed an intimate virtual gathering of 3–6 project managers to air their frustrations and problem-solve any COVID-19 team or project management-related issues.

As the COVID-19 Research Initiative advanced, project managers became responsible for an increasing number of deliverables for their teams. In addition, these priorities became intertwined with new responsibilities when CTSI was awarded a 5-year $23 M Clinical and Translational Science Award (NIH CTSA; 2UL1 TR001436). Signs of stress increased as CTSI staff began shifting their focus to the new award while carrying out their COVID-19 project management responsibilities.

#### 4.2.2 Project manager listening sessions: team science challenges and best practices for future research initiatives

CTSI leadership responded to these signs of stress by holding multiple listening sessions during the week of July 13^th^, 2020, where project managers expressed their concerns and frustrations, and offered suggestions. Afterward, the project managers team met with the director of CTSI and jointly discussed their concerns and brainstormed potential solutions. In general, these issues fell into two categories: (A) project manager concerns, e.g., adequate project management training, role definitions, task preparedness, and project manager bandwidth; and (B) COVID-19 Research Team concerns, e.g., leadership weaknesses, insufficient research experience, and gaps in expertise among team members (see Table 4). This information led to the formation of best practices for the future, some targeted interventions, and helped illustrate the general barriers that staff and faculty experienced while supporting the initiative. In addition, the exercise led to some relief from daily responsibilities and activities of the initiative, and more scrutiny of the burden/value ratio when considering new project manager responsibilities.

**Table 4:** Summary of concerns discussed by project managers during multiple listening sessions, and suggestions developed during a brainstorming session with project managers and the director of CTSI.

Reported Issues	Suggested Solutions
Project Managers Unprepared Some project managers were assigned to teams after the team had already been meeting, and the project managers felt unprepared as the group looked to them for direction and answers. Project managers felt overwhelmed with prioritizing team needs and trying to solve team barriers.	For future situations like the pandemic, the CTSI Project Management Office could write standard operating procedures and store all the resources so that future projects can refer to these during training and preparation. Cross-train the CTSI staff for dual-purpose roles in preparation for an emergency.
Team Leadership & Grant Writing Experience No orientation for team leaders, and expectations for team leaders were not provided. In some cases, grant-writing and grant-submission experience was lacking by both team leaders and team members. The institutional process for grant submissions is different across partner institutions, and this was a barrier for those unfamiliar with procedures at other institutions. There were assumptions that the investigators would naturally take the lead, but in fact, some project managers were made “leaders” by default. Although project managers were provided grant basics and grant submission training, the accelerated pace of the initiative posed a great challenge to incorporate the tidal wave of new terminology and processes.	Provide clarification of the project manager role at the beginning of the initiative. Possibly hire a resource for grant writing assistance for use across all teams. Once a team leader is established, bring in a seasoned “Sr. Project Manager” to help brief the new team on the team leader role. The role of the project manager is not to write the grant, but to lead the team to sources for training.
Research Experience Some teams that were comprised of novice researchers and junior faculty had little experience with the academic research process and team science best practices. Some team members did not have any experience with, or adequate understanding of the Ensemble process.	It would be encouraging for the teams to have CTSI leaders or seasoned researchers join in the meetings periodically. Also, create a seminar session for teams to learn more about the Ensemble process and know what is expected of team members.
Anxiety From Facing Many Unknowns The Planning Committee & Project Managers had no idea how long the pandemic would last. Moreover, CTSI personnel had no idea how long they would continue in their COVID-19 roles. This weighed on the minds of project managers as CTSI gradually returned to normal daily activities, and bandwidth shrank among the project managers.	The use of *ad hoc* project management could be a way to move forward with some teams that are slowing down, and require less support. Each project manager needs to evaluate their team’s status to help prioritize what resources are needed and determine frequency of meetings.

#### 4.2.3 Institutional support for F&MCW faculty, staff, and frontline healthcare workers

CTSI faculty and staff were also encouraged to take advantage of several institutional support services provided by F&MCW. One of these support services included collaboration between the Psychiatry and Behavioral Medicine Department and Human Resources. Together, they created virtual peer support group sessions for faculty and staff, led by trained facilitators. These support activities were an extension of the groundwork achieved by the COVID-19 team that had created the Building Resilience Virtually (BRaVe) Clinic (43).

### 4.3 Team science performance factors

#### 4.3.1 Team leadership

The importance of leadership ability and style in team science settings has been discussed extensively in the literature (27, 32, 33, 36, 37, 58, 68). Team leaders must attend to a large array of responsibilities to effectively lead a group—especially in multidisciplinary and multi-institutional teams. Moreover, our COVID-19 team leaders faced even greater leadership challenges as a result of the urgency of the COVID-19 Research Initiative and the necessity to lead teams in a totally virtual work environment. For some leaders, it was also their first time leading a multidisciplinary research team, and these factors influenced the success of some teams. Project managers reported an apparent connection between leadership style and capability, and team success. This included the team leader’s ability to (1) organize and communicate team activities and deliverables, (2) encourage participation from all team members, and (3) coach others with less research experience. As described in Table 4, project managers reported that in some cases, no team member emerged as a leader, causing an overreliance on the project manager to lead team activities. In some cases, the opposite was true, in which an investigator “grabbed” the team leader role. In at least two cases of this “power-grab,” team member attendance plummeted after these leaders began monopolizing team discussion. Both teams retired soon after forming. Collectively, most team leaders in the initiative helped achieve significant milestones with their teams. Reports on negative team leader experiences were limited, and these were similar to those previously described in the literature regarding leadership experience, styles, ability, and training.

#### 4.3.2 Team gaps in research experience and disparate institutional grant submission processes

In addition to leadership style and experience, project managers identified other gaps in experience among team members as potential influencers of team success. For example, some teams lacked members with experience in the grant writing or grant submission process. These deficits were addressed in different ways. One team recruited a new member with the necessary experience, whereas a few teams simply leaned on the project manager to guide the grant writing and submission process. Overall, grant writing abilities were a significant roadblock for several teams. The multi-institutional participation on teams also caused challenges, due to important differences in institutional grant submission policies, procedures, and submission software.

As the initiative moved forward, teams also identified gaps in scientific expertise. Project managers and team leaders worked to fill these gaps through basic networking practices. However, CTSI also developed a systematic process in which requests for specific expertise were forwarded to the Leadership Advisory Committee, who helped identify and place team members.

#### 4.3.3 Team challenges due to institutional impediments, conflicting priorities for investigators, and low bandwidth

Team success was impacted by both institutional and investigator priorities, which sometimes made team participation more challenging. For example, the reason cited most often by investigators for leaving the initiative was “lack of bandwidth.” Departing investigators indicated the need to attend to (1) departmental priorities, (2) personal research goals, and (3) clinical schedules. Teaching responsibilities also caused attrition. In fact, we saw the retirement of four teams in the month immediately following the beginning of the fall semester. These types of institutional and investigator priorities that sometimes pit individual career needs against multidisciplinary team participation have been previously highlighted in other articles (32, 33). In addition, institutional policy decisions sometimes had the unintended effect of blocking the ability to conduct certain research. For example, we encountered new institutional policies in response to COVID-19 that made laboratory research difficult. This included a lack of PPE for research laboratory staff because of prioritizing the use of PPE in the clinical enterprise. In some cases, the absence of appropriate biosafety equipment also prevented direct study of COVID-19 biospecimens, or use of the SARS-CoV-2 virus. F&MCW was not alone in this challenge: Similar difficulties were highlighted at other prominent research centers, as well (69). These decisions, although necessary to protect research staff, impacted the participation by some investigators in the initiative.

#### 4.3.4 Funding obstacles are a key team science challenge

In a survey of 651 faculty researchers in the State University of New York System, lack of funding was identified by 280 (43%) survey participants as the top-ranked barrier to effective research collaboration (70). This was also a key challenge for some investigators in our initiative, illustrated by project manager observations (Table 4) and investigator exit responses (Table 3).

Funding challenges were further exacerbated by barriers within grant-making agencies. At one point in the early stages of the pandemic, CTSI was notified that NIH had more proposal submissions than time to review them. News of the backlog was not encouraging, and understandably, maintaining team enthusiasm could be challenging when the prospects for funding appeared less than favorable. Most team members had to consider individual career needs, and certainly, this contributed to some team members leaving the initiative and causing some teams to retire prematurely. This challenge could be addressed in future public health crises by creating an emergency funding mechanism for research initiatives. For example, an endowment could be established by regional academic medical institutions to create an emergency fund in which certain crisis events triggered the availability of funds for intramural or regional grant competitions. Some of these research funds could target local health challenges caused by a national public health emergency. For example, research funds could be used to solve problems exacerbated by SDOH that are specific to local communities, vulnerable populations, or geographic regions. Of course, the capacity to invest in future emergency funding mechanisms is not a simple act, and is complicated by the current fiscal challenges faced by most academic medical research institutions (71). Despite the potential disappointment from funding challenges, many COVID-19 teams persisted and achieved significant milestones.

### 4.4 Suggestions for team-building activities, interventions, and supports to address team science challenges

Although the COVID-19 Research Initiative was successful in forming many teams, we saw the gradual retirement of teams throughout the first 8 months of the initiative. More importantly, several teams ended quite early, with seven teams retiring during the first 5 weeks of existence. In reviewing the results of the initiative, we identified several approaches that could help build and sustain teams for future initiatives. Some project managers suggested that in the future, CTSI should provide teams with a process for selecting leaders, as well as guidelines and expectations of leaders, and create lunch and learn sessions for new leaders.

During our initiative, some teams received valuable guidance from a small collection of 4–5 seasoned translational researchers from CTSI. However, these senior investigators were also leaders of their own teams, so their bandwidth to help other teams was limited. For future research initiatives, we recommend assigning seasoned translational researchers or experienced team leaders to each team during the critical early stages of team development. They could provide guidance that might include (1) assistance with project design, (2) identification of available research resources, or (3) awareness of standard team science principles and processes. They could even provide structured mentorship for junior leaders. Abbreviated workshops on team science could also be provided to help bolster team leadership skills.

We also suggest providing written tools for team activities that may help project managers facilitate team science collaboration. Some team members were frustrated or confused by the nucleation steps and the Ensemble process. Therefore, we suggest creating “signposts” for the “roadmap,” to be visual tool indicating the current “location” of the investigator and the next milestone. In addition, we recommend that the leadership of large research initiatives attend individual research team meetings to (1) “take the pulse” of the overall initiative membership, (2) provide guidance, and (3) continually reinforce the instructions from project managers. Moreover, we recommend building in time for structured investigator listening sessions, so that team members can express challenges, air frustrations, and develop strategies to overcome common barriers. We also recommend developing interventions to respond to immediate challenges that are identified by project managers. Early interventions might have helped bridge difficult periods for teams in this initiative.

### 4.5 Adapting the CTSI Ensemble model for COVID-19 team formation and process

#### 4.5.1 Debut of the CTSI Ensemble program

When the COVID-19 Research Initiative began (March 2020), the CTSI Ensemble Program was fairly new. The program debuted in June 2019 with the formation of 12 Pre-Ensembles; these are the precursor teams that develop over 3–4 months and then compete for Ensemble funding. This initial RFA cycle resulted in the approval of funding for the first 10 Ensembles, with a $50 K line of credit per Ensemble. Since then, the Ensemble Program has released several cycles of funding, leading to the formation of 110 Pre-Ensembles and a total of 28 funded Ensembles. These Ensembles have enjoyed broad multidisciplinary expertise, multi-institutional integration, and significant community partnerships. Moreover, these teams were built for long-term growth —Most teams have not only persisted, but have also flourished for several years after exhausting their CTSI funding. Several Ensembles have published the results of preliminary research, submitted grant proposals, received awards, and are conducting human research studies with community partners. These results began to accumulate following a brief pause in research activity caused by the pandemic. Thus, the productivity generated by teams formed through the COVID-19 Research Initiative gave CTSI the first positive indication of this novel model’s potential.

#### 4.5.2 Similarities between COVID-19 teams and current CTSI ensemble program teams

The adaptation of the Ensemble model to the COVID-19 Research Initiative provided a successful template to create extremely robust multidisciplinary teams, with representation on most teams from the three domains described by the Mutually Learning Tri-lateral Ecosystem (Section 2.4). In general terms, we found that the distribution of Ensemble-defined roles (e.g., basic scientists, community/population science researchers, clinical investigators, hospital system representatives, community member, patients, etc.) was similar between the teams of the COVID-19 Research Initiative and the current composition of teams in the Ensemble Program. However, participation was lower for the “community member” role and “patient representative” role on COVID-19 teams. Although some teams partnered with community organizations, many COVID-19 teams concentrated their early efforts on recruiting the clinical, basic science, and epidemiological expertise, rather than focusing on individual community and patient representatives. In addition, patient and community members were likely to be focused on their own urgent priorities, or had difficulties with participating virtually, as was the case with other team members.

Another important similarity we observed between the COVID-19 teams and the Ensemble Program teams was the fundamental “readiness” for adapting and evolving, as necessary. This Ensemble fundamental was especially vital to the COVID-19 teams since team members needed to adapt team goals and corresponding team composition to the quickly shifting research priorities and barriers of the COVID-19 pandemic. This was evident from the addition of new team members that were needed for evolving research targets, as well as the movement of investigators between teams for aligning expertise. This was also demonstrated by the modifications to team names, and the seamless process of subdividing or merging of teams.

#### 4.5.3 Addressing the challenges of using a novel, atypical approach for multidisciplinary team collaboration

Although the adaption of the Ensemble model to the COVID-19 Research Initiative was quite successful, some observations provided us with ideas for potential changes in the event that a similar research initiative is needed in the future. For example, at the time of the pandemic, the Ensemble model was in its infancy and only a small number of investigators were aware of the concept. It is likely that the novelty of this approach was received differently by investigators, depending on their level of experience with C&TR and team science fundamentals. To be certain, the Ensemble concept is vastly different.

For example, the unique process and requirements for team member collaboration within the Ensemble Program are quite different from traditional team research approaches. By design, a newly formed Pre-Ensemble typically requires a significant investment of time to develop a shared vision and tangible goals, prior to conducting research and embarking on a search for extramural funding. It is also necessary for team members to develop team science skills for multidisciplinary collaboration. All this takes more team member dialogue and time than the typical preparation to submit a standard, single- or multiple-PI grant proposal.

Some investigators in the COVID-19 Research Initiative may have found this collaboration process to be too abstract, and were frustrated with a substantial time commitment that lacked immediate, tangible research projects and grant proposals, given the immediacy of the pandemic crisis. For example, in an exit interview, one investigator responded, “(I) did not like the group structure—another meeting to set up a meeting, etc. Seemed like a waste of time.” As mentioned earlier, providing team-building tools with more guidance on the collaborative steps and interim process goals could help increase success of teams during a future public health crisis. Moreover, the knowledge gained from 5 years’ experience with the CTSI Ensemble Program provides a robust collection of exemplary models for success and lessons learned to improve the design and execution of future research initiatives like the COVID-19 Research Initiative.

## 5 Conclusion

### 5.1 CTSI groundwork for a successful regional research initiative

The COVID-19 Research Initiative demonstrated that the CTSI consortium of eight partner organizations could come together quickly to plan, develop, and execute a successful regional research initiative within days of the “Call to Action.” However, the groundwork prior to the pandemic cannot be overstated. Before March 2020, CTSI worked for more than a decade integrating the eight partner institutions and building this hub’s collaborative platform. This included the implementation of multiple CTSA programs to develop multi-institutional, multidisciplinary C&TR. At the same time, CTSI developed meaningful partnerships with many community organizations, especially ecumenical organizations that serve the City of Milwaukee’s vulnerable populations. All of these CTSI programs and partnerships contributed to a fertile environment when the time came to nucleate investigators into COVID-19 research teams. CTSI is also a nimble entity by design that is constantly evolving as it builds novel programs and alters course intentionally as a result of real-time feedback and data. The existence of this culture allowed CTSI to quickly adapt to the needs of this initiative. Very importantly, previous CTSI groundwork included the development of two novel models (Section 2.4, the Mutually Learning Tri-lateral Ecosystem and the CTSI Ensemble), which provided the initiative with the fundamentals for successful team formation and operation.

### 5.2 CTSI core values and growth

Throughout the COVID-19 Research Initiative, CTSI faculty and staff brought their passion and core values for “making the best medicine better, and delivering more solutions, more quickly, to patients and communities.” Not surprisingly, the work of CTSI faculty and staff during the initial 6 months of the initiative was frequently accompanied by significant stressors. However, CTSI was quick to provide support to staff, and as a result of the pandemic, CTSI continues to grow as an organization that prioritizes active listening and brainstorming to collectively address all challenges. A core principle underlying all CTSI operations and projects is a conviction to use every result to inform course corrections and improve processes. The COVID-19 Research Initiative was a success because of this principle.

### 5.3 The impact of other CTSA hubs on COVID-19 C&TR

The impact of CTSI on COVID-19 research in Southeast Wisconsin reflects a common theme echoed by the other CTSA academic hubs across the U.S. In a survey of 60 CTSA hubs, Jayaweera et al. found that many of these CTSA hubs played a significant role at the institutional level for prioritizing research projects, establishing institutional policies in the clinical trials office and IRB office, providing COVID-19 funding, and execution of C&TR (69). One of the key takeaways from the survey was that CTSA awards “played a major role in the current pandemic and should be empowered to do so in the future.”

### 5.4 Translational workforce development: team science experience and skills

The attraction of joining a high-profile COVID-19 Research Initiative provided a compelling, if not exciting, opportunity to gain experience in multidisciplinary/multi-institutional C&TR. The initiative attracted a broad collection of investigators and stakeholders with experience levels ranging from novice to seasoned researchers. Many junior investigators gained team science-guided C&TR experience for the first time, including skills and processes for forming teams, leading others, multi-institutional collaborating, writing or submitting grants, and team science participation in the critical appraisal of research ideas. In addition, CTSI staff were afforded an invaluable experience from being embedded in C&TR teams. They developed new knowledge and skills, including the use of team science principles for the project management of multidisciplinary, multi-institutional teams. Many project managers learned to interpret RFA requirements, navigate institutional research policies, and manage grant proposal development. In some cases, they had to provide leadership or facilitate difficult conversations to determine the fate of a team. In retrospect, it is hard to imagine a more immersive experience to build these translational workforce skills.

### 5.5 A model for the future

Several publications [both pre-COVID-19 pandemic (72, 73) and post-COVID-19 pandemic (74–79)] have proposed a variety of strategies for accelerating research during a public health emergency. Some of these strategies include (1) developing a process for activating a rapid research response, (2) ensuring adequate depth of investigator rosters and bandwidth for clinicians, (3) involving affected communities and understanding their concerns, (4) creating rapid IRB review processes that integrate multi-institutional IRB review boards, (5) creating the infrastructure for rapid data collection and sharing, and (6) developing a process to identify the most pertinent research questions and rapid funding. We have shown that the COVID-19 Research Initiative was developed and executed using several CTSI principles, models, approaches, and processes that were similar to these recommended strategies.

While there have been several publications on models and programs for conducting rapid research response in the midst of a public health emergency, we found no publications that reported on the development, execution, and lessons learned from a real-life rapid research response, like the COVID-19 Research Initiative. The experience of the initiative provided a wealth of qualitative data to improve and expand on the successes of this regional initiative and prepare for conducting C&TR in future global public health emergencies. It is now really up to us all to apply the lessons learned, and capitalize on the innovation that changed the way we respond to such threats in patient-centered research, clinical practice, public health surveillance and action, and population health.

## Data Availability

The raw data supporting the conclusions of this article will be made available by the authors, without undue reservation.
