# Supplementary material for: Public health emergency accelerated research response—the Clinical and Translational Science Institute of Southeast Wisconsin COVID-19 research initiative
Source: Front Public Health. 2025 May 9;13:1529121. doi: 10.3389/fpubh.2025.1529121 (PMC12098277; doi:10.3389/fpubh.2025.1529121)
Supplement: Supplementary file 1 [file Data_Sheet_1.pdf]

Supplement 4: Investigator Roadmap with Team Implementation Steps

CTSI COVID-19 Research Initiative: Pre-Ensemble Nucleating Plan (Draft v2.0)						
**Tuesday, 4/7**	**Wednesday, 4/8**	**Thursday, 4/9**	**Friday, 4/10**	**Friday 4/10 – Tuesday 4/14**	**Wednesday, 4/15**	**Thursday, 4/16**
**Receipt of Invitation from CTSI to Self-Select into Research Teams**	**12:00 pm:**<br>**Virtual Town Hall Meeting I**	**9: 00 am:**<br>**Deadline for Self-Selection Per Invitation**<br><br>**11:00 am:**<br>**Deadline for 1st Assignment**	**8:00 am – 5:00 pm:**<br>**Enter Microsoft Teams Environment for COVID-19 Pre-Ensemble Team Introductions**<br>**(Team meeting times will vary based on the availability of team members)**	**Research Project Development (Virtual within Microsoft Office Teams Environment and Offline as needed)**	**2:00 pm:**<br>**Deadline 2nd Assignment**	**Next Steps To Be Determined by Teams**
• Invitation to either Virtual Town Hall Meetings on Wednesday, April 8th at 12:00 pm or 4:00 pm<br>• Per invitation, indicate research interest and self-select into research group of interest ASAP (deadline is Thursday, 4/9 by 9:00 am)	**Attend Virtual Town Hall Meeting**<br><br>**Meeting Agenda:**<br>• Call to Action<br>• Process to Nucleate around COVID-19 Research Questions<br>• 5-minute Primer on Pre-Ensembling – i.e, a process, not a grant-writing activity; leads to products (e.g., protocols/grants)<br>• Concise Research Question List<br><br>**4:00 pm:**<br>**Virtual Town Hall Meeting II**<br><br>**Attend Virtual Town Hall Meeting**<br><br>• See Meeting Agenda above	• Complete and submit Self-Selection choice(s)<br>• Upon receipt of results, CTSI Project Mgr. will make assignments to a Microsoft Office (MS) Teams Group, per research topic choice<br>• Submit SINGLE Slide PPT describing Research Interests and Resources<br><br>1st Assignment:<br>• Each investigator creates a SINGLE Slide PPT describing Research Interests & Resources	• Establish Research Team Lead(s)<br>• Future Meetings will be determined<br>• SINGLE PPT Slides will be reviewed and preliminary discussions will occur within Microsoft Teams environment<br>• Each team will have a CTSI Project Manager assigned to assist with project development and submission<br>• Post-meeting, consider research project strategy (specific aims, narrative/summary, etc.)	• Discussion on research projects (research ideas, opinions, human and technological resources etc)<br>• Work with CTSI Project Manager and research team members to continue describing unmet medical need, background, scientific and technical resources and potential products such grants, clinical trials, devices etc.<br>• Discuss project support needs (biostats., writing, pre-submission activities, etc.)<br>• Strategize on next steps and discuss timelines	• Submit research project summary (e.g., Significance Page describing Unmet Medical Need, Background & Scientific, Technical Resources, Expertise, Potential Products, etc.)<br>• Discuss and plan next steps and timelines for finalizing research projects	• Focus initial efforts/resource s on High Priority Research Questions<br>• COVID-19 Pre-Ensemble Microsoft Teams continue to meet & refine projects

Supplement 4: Investigator Roadmap (Continued)

**Deliverable:**<br>• Select research interest topic and submit by Thursday 4/9/20, 9:00 am	**Deliverable:**<br>• Review of meeting agenda and attend one of the Virtual Townhall Meetings on Thursday, 4/9/20	**Deliverable:**<br>• Due date for research topic self-selection<br>• Create a single ppt slide with research interest/resources and submit<br>• Submit Single ppt slide describing research interest and resources<br>• Prepare to participate and share ppt and Significance content/summary/ narrative	**Deliverable:**<br>• Enter Microsoft Teams environment as assigned and meet members and Project Manager for your team<br>• Participate in discussion, --planning, share ppt content, Significance content, etc.<br>• Choose Team Lead<br>• Participate in next steps and timelines discussion	**Deliverable:**<br>• Participate in group Microsoft Teams and offline research development meetings<br>• Continue to develop research ideas with team members<br>• Connect with Project Manager as needed to stay on the same page with team lead/members, and on track with timelines<br>• Teams to discuss project needs and communicate these with team members and Project Managers<br>• Strategize and write—be clear about your assignments/roles	**Deliverable:**<br>• Submit research project narrative—Significance Page describing Unmet Medical Need Background & Scientific, Technical Resources, Expertise, Potential Products is due<br>• Participate with Team activities, meetings, decision-making; and participate/interact with Project Manager	**Deliverable:**<br>• Next Steps to be determined by Teams
