# Supplementary material for: Public health emergency accelerated research response—the Clinical and Translational Science Institute of Southeast Wisconsin COVID-19 research initiative
Source: Front Public Health. 2025 May 9;13:1529121. doi: 10.3389/fpubh.2025.1529121 (PMC12098277; doi:10.3389/fpubh.2025.1529121)
Supplement: Supplementary file 2 [file Data_Sheet_2.pdf]

Supplement 5: Team Charter Example

Team Research Question:<br><br>**Collect feasibility data for conduct of future innovative Phase 1/2 clinical trials using existing drugs and biologics.**	Team Section:<br><br>**Treatment and Vaccine**
**Team Purpose & Patient Benefit:**<br><br>• A Phase II/III Randomized Controlled Trial of Salmeterol vs. Placebo for Inpatients with COVID-19 (The Salmeterol Trial)<br>• A Phase II/III Randomized Controlled Trial of Salmeterol vs. Placebo for Outpatients with COVID-19 (The Salmeterol Outpatient Trial)	**Potential Team Products:** NIH grant, multicenter clinical trial through either SIREN, MARCH, or TIN. Patent application
**Background of Unmet Medical Need:** No approved drugs for treatment of COVID-19	**Active Team Members:** 5-7
**Team Member Roles:** Clinical Investigator, Basic Science Investigator	**Submitted Grant (Date/Agency):** NA<br>**Potential Grant (Date/Agency):** NINDS administrative supplement
**Expertise Needed:** Biostats, CTO, IDS pharmacy. All accessed and available	BSL3 Needed: No<br>COVID-19 Mice Needed: No<br>COVID-19 Biospecimens Needed: Yes<br>Other Resources Needed: Drug being sourced from GSK; looking for placebo supplier, School of Pharmacy assisting

Potential Project Details and Specific Aims:

Inpatient Trial:

Primary Aim

Determine the change in respiratory SOFA score (PaO2/FiO2) at 72 hours during hospitalization in adults aged ≥18 years old with a presumptive positive laboratory test for COVID-19 admitted to the hospital ward or intensive care unit (ICU) for COVID-19 symptoms treated with SEREVENT Inhalation Aerosol (salmeterol 50 mcg) 1 puff PO BID X 14 days or until death or hospital discharge versus placebo

Secondary Aims

Determine the daily Sequential Organ Failure Assessment (SOFA) scores to day 14, death or hospital discharge, whichever comes first, ventilator free days, vasopressor free days utilization of Bilevel Positive Airway Pressure (BiPAP), high flow nasal cannula, mechanical ventilation, or extracorporeal membranous oxygenation (ECMO) utilization, the percentage of subjects with a severity rating on a 7-point ordinal scale at day 14, hospital length of stay, rate of ICU admission in patients initially admitted to the ward, length of ICU stay, rate of intubation, and survival to hospital discharge in adults aged ≥18 years old with a presumptive positive laboratory test for COVID-19 admitted to the hospital ward or ICU for treatment of COVID-19 symptoms.

Outpatient Trial:

Primary Aim

Determine the hospitalization rate in adults aged ≥18 years old with a positive laboratory test for COVID-19 discharged from an emergency department or screening clinic for COVID-19 symptoms treated with SEREVENT Inhalation Aerosol (salmeterol 50 mcg) 1 puff PO BID X 15 days from randomization or until hospitalization versus placebo

Secondary Aims

Determine the subjective improvement in dyspnea as measured by the dyspnea PROMIS measures at day 0, 4, and 10, time to symptom resolution, Short Form 12 Health Survey at days 0, 4, and 10, daily maximum temperature, number of emergency department or clinic presentations within 15 days, and disease severity rating on the *7-point ordinal scale* at 15 days
