# Supplementary figures and images for: Public health emergency accelerated research response—the Clinical and Translational Science Institute of Southeast Wisconsin COVID-19 research initiative
Source: Front Public Health. 2025 May 9;13:1529121. doi: 10.3389/fpubh.2025.1529121 (PMC12098277; doi:10.3389/fpubh.2025.1529121)

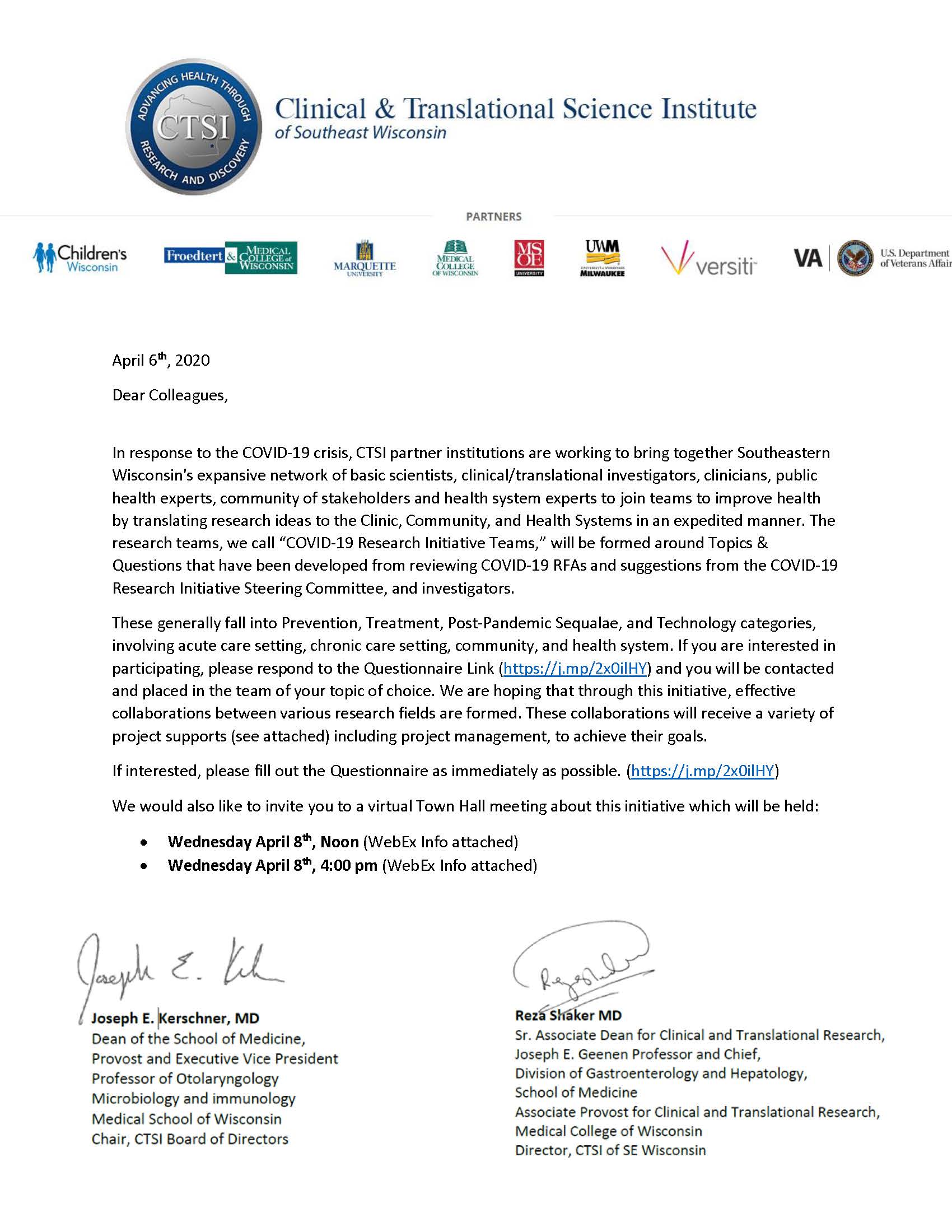

Supplement: Supplementary file 3 [file Image_1.jpeg]
