# Supplementary material for: Public health emergency accelerated research response—the Clinical and Translational Science Institute of Southeast Wisconsin COVID-19 research initiative
Source: Front Public Health. 2025 May 9;13:1529121. doi: 10.3389/fpubh.2025.1529121 (PMC12098277; doi:10.3389/fpubh.2025.1529121)
Supplement: Supplementary file 4 [file Table_1.docx]

Supplementary Material

**Supplement 1: COVID-19 Research Initiative Planning Committee.**

| **Tanya Aigner**, Project Manager, CTSI Community Engagement; CTSI, Medical College of Wisconsin |
| --- |
| **Mike Anello, BA**; Program Director I; COVID-19 Research Initiative of SE WI Lead Project Manager, Ensemble Program Lead Project Manager; Multidisciplinary Team Science Workshop Co-Director; CTSI, Medical College of Wisconsin |
| **Memory Bacon**, CTSI Academy Program Manager; CTSI, Medical College of Wisconsin |
| **Ciara Davis, MBA**; Program Director I, Governance and Administration; CTSI, Medical College of Wisconsin |
| **Rebecca Gasper, MPH**; CTSI Academy Education Coordinator; CTSI, Medical College of Wisconsin |
| **Orsolya Garrison, DrPH, MPH**; Assistant Professor, Director of Operations; CTSI, Medical College of Wisconsin |
| **Amit Gode; MBBS, MPH**; Assistant Professor, Director of Operations; CTSI, Medical College of Wisconsin |
| **Angelina Holtz; Project Manager**, CTSI Community Engagement; CTSI, Medical College of Wisconsin |
| **Dessie Levy, PhD**; Assistant Professor, CTSI Community Engagement; CTSI, Medical College of Wisconsin |
| **Renee McCoy**; Program Director I, CTSI Pilot Translational and Clinical Studies Program; CTSI, Medical College of Wisconsin |
| **Dian Mitrayani, PhD**; Program Manager II, CTSI Evaluation and Continuous Improvement; CTSI, Medical College of Wisconsin |
| **Ramez Rashid, PhD**; Director of Evaluation and Quality CTSI Evaluation and Continuous Improvement; CTSI, Medical College of Wisconsin |
| **Reza Shaker, MD**; Associate Provost for Clinical and Translational Research; Senior Associate Dean and Director of Clinical and Translational Science Institute of Southeast Wisconsin; Joseph E. Geenen Professor and Chief of Gastroenterology and Hepatology; Director of Digestive Disease Center; Froedtert Hospital & Medical College of Wisconsin |
| **Shankar Srinivasan, PhD**; Assistant Professor; Director, Clinical Trials Office and Translational Research Unit; CTSI, Froedtert Hospital & Medical College of Wisconsin |
| **Louann Sullivan**; Project Coordinator; CTSI Community Engagement; CTSI, Medical College of Wisconsin |
| **Bradley Taylor, MBA**; Chief Research Informatics Officer; CTSI, Froedtert Hospital & Medical College of Wisconsin |
| **Doriel Ward, PhD, MPH**; Chief Administrative Officer for Clinical & Translational Research; Assistant Dean of Clinical and Translational Research, School of Medicine; Executive Director of CTSI; Assistant Provost of Clinical and Translational Research, School of Medicine; Medical College of Wisconsin |
| **Katherine J. Woods, MS, CCRC**; Business Analyst IV, Data Operations Manager, Biostatistics, Epidemiology, and Research Design; CTSI, Medical College of Wisconsin |
| **David Zimmerman, MBA, MPH**; Senior Project Manager, Ensemble Program; CTSI, Medical College of Wisconsin |

**Supplement 2: COVID-19 Research Initiative Leadership Advisory Committee**

| **Members of the Council of Collaboration (2020)** | **Title, Credentials, Department, Institutions** |
| --- | --- |
| **Stephen Hargarten, MD, MPH, Professor of Emergency Medicine** | Founding Director of the Comprehensive Injury Center; Senior Science and Policy Advisor; Froedtert Hospital & Medical College of Wisconsin |
| **Ivor Benjamin, MD, FAHA, FACC, Professor of Medicine** | Professor, Medicine, Physiology, Pharmacology and Toxicology, Cell Biology, Neurobiology and Anatomy, and Surgery; Director of the Cardiovascular Center; Co-Director of the NIH T32 Postdoctoral Fellowship in Cardiovascular Sciences and Co-Leader of the Cardiovascular Center’s Signature Program in Precision Cardiovascular Medicine; Froedtert Hospital & Medical College of Wisconsin |
| **Leonard Egede, MD, MS, Professor of Medicine** | Inaugural Milwaukee Community Chair in Health Equity Research at MCW; Division Chief of General Internal Medicine; Director of the Center for Advancing Population Science (CAPS); Director of Medical college of Wisconsin CTSI KL2 Program; Director of MCW CTSI Master’s in Clinical & Translational Research; Froedtert Hospital & Medical College of Wisconsin |
| **Shi-Jiang Li, PhD, Professor of Biophysics** | Professor Emeritus; Medical College of Wisconsin |
| **John Meurer, MD, MBA, Professor of Pediatrics** | Professor of Pediatrics and Community Health; Director of the Institute for Health & Equity; Froedtert Hospital & Medical College of Wisconsin |
| **George MacKinnon, PhD, MS, RPh, FASHP, FNAP, Professor of Pharmacy** | Founding Dean of the School of Pharmacy and Program Development; Froedtert Hospital & Medical College of Wisconsin |
| **Ravindra Misra, PhD, Dean, Professor of Biochemistry** | Dean of Graduate School of Biomedical Sciences; Medical College of Wisconsin |
| **Hallgeir Rui, MD, PhD, Professor of Pathology** | Wisconsin Breast Cancer Showhouse (WBCS) Endowed Professor of Breast Cancer Research; Director of MCW Tissue Bank; Professor and Vice Chair of Research, Pathology; Medical College of Wisconsin |
| **Reza Shaker, MD, Professor of Gastroenterology** | Associate Provost for Clinical and Translational Research; Senior Associate Dean and Director of Clinical and Translational Science Institute of Southeast Wisconsin; Joseph E. Geenen Professor and Chief of Gastroenterology and Hepatology; Director of Digestive Disease Center; Froedtert Hospital & Medical College of Wisconsin |
| **Raul Urrutia, MD, Professor of General Surgery** | Linda T. and John A. Mellowes Director of Center for Genomic Sciences and Precision Medicine; Medical College of Wisconsin |
| **Doriel Ward, PhD, MPH, Professor** | Chief Administrative Officer for Clinical & Translational Research; Assistant Dean of Clinical and Translational Research, School of Medicine; Executive Director of CTSI; Assistant Provost of Clinical and Translational Research, School of Medicine; Medical College of Wisconsin |
| **Ming You, MD, PhD, Professor of Pharmacology and Toxicology** | Associate Provost, Professor, PhD, Pharmacology and Toxicology; Medical College of Wisconsin |
| **CTSI Executive Committee (2020)** | **Title, Credentials, Department, Institutions** |
| **Mark T Harris, PhD, MS Professor of Geosciences** | Vice Provost for Research, Department of Geosciences; University of Wisconsin-Milwaukee |
| **Jeanne Hossenlopp, PhD, Professor of Chemistry** | Vice Provost for Research & Innovation; Marquette University |
| **Sheku Kamara, MSE, BSMEng Dean of Applied Research** | Dean of Applied Research at the Applied Technology Center (ATC); Milwaukee School of Engineering |
| **Paula Papanek, PhD, MPT, LAT, ATC, FACSM, CEEAA, Professor of Exercise Science** | Director of Program in Exercise Science; Director of Graduate Program in Sports and Exercise Analytics; Marquette University |
| **Reza Shaker, MD, Professor of Gastroenterology** | Associate Provost for Clinical and Translational Research; Senior Associate Dean and Director of Clinical and Translational Science Institute of Southeast Wisconsin; Joseph E. Geenen Professor and Chief of Gastroenterology and Hepatology; Director of Digestive Disease Center; Froedtert Hospital & Medical College of Wisconsin |
| **Ward, Doriel, PhD, MPH, Professor** | Chief Administrative Officer for Clinical & Translational Research; Assistant Dean of Clinical and Translational Research, School of Medicine; Executive Director of CTSI; Assistant Provost of Clinical and Translational Research, School of Medicine; Medical College of Wisconsin |
| **Gilbert White, MD, Professor of Hematology and Oncology** | Director of Research at Versiti Blood Research Institute Foundation, Inc., Executive Vice President & Chief Scientific Officer, Versiti Blood Center; Medical College of Wisconsin |
| **Calvin Williams, MD, PhD, Professor of Pediatrics, Microbiology, and Immunology** | Associate Dean of Research; Associate Director of Medical Scientist Training Program; Chief Scientific Officer of Children's Wisconsin Research Institute; Section Chief of Pediatric Rheumatology; Children's Wisconsin, Medical College of Wisconsin |
| **CTSI Team Science-Guided Integrated Clinical and Research Ensemble Review Committee** | **Title, Credentials, Department, Institutions** |
| **Roy Silverstein, MD, Professor of Hematology and Oncology** | Linda and John Mellowes Professor and Chair of the Department of Medicine; Associate Director of the Linda T. and John A. Mellowes Center for Genomic Sciences and Precision Medicine; Froedtert Hospital & Medical College of Wisconsin |
| **Ravindra Misra, PhD, Professor of Biochemistry** | Dean of Graduate School of Biomedical Sciences; Medical College of Wisconsin |
| **Christopher Schultz, MD, FACR, FASTRO, Professor of Radiation Oncology** | Bernard & Miriam Peck Family Professor and Chair of Radiation Oncology; Froedtert Hospital & Medical College of Wisconsin |
| **Jon Lehrmann, MD, Professor of Psychiatry & Behavioral Medicine** | Charles E. Kubly Professor and Chair in Psychiatry & Behavioral Medicine; Froedtert Hospital & Medical College of Wisconsin |
| **Balaraman Kalyanaraman, PhD, Professor of Biophysics** | Harry R. & Angeline E. Quadracci Professor in Parkinson’s Research; Chair of the Department of Biophysics; Medical College of Wisconsin |
| **Jonathon Truwit, MD, Professor Emeritus, Pulmonary Medicine** | Chief Medical Officer & Senior Associate Dean for Quality; Froedtert Hospital & Medical College of Wisconsin |
| **Cynthia Lien, MD, Professor of Anesthesiology** | Professor and Chair of the Department of Anesthesiology at Froedtert Hospital East; Froedtert Hospital & Medical College of Wisconsin |
| **Reza Shaker, MD, Professor of Gastroenterology and Hepatology** | Associate Provost for Clinical and Translational Research; Senior Associate Dean and Director of Clinical and Translational Science Institute of Southeast Wisconsin; Joseph E. Geenen Professor and Chief of Gastroenterology and Hepatology; Director of Digestive Disease Center; Froedtert Hospital & Medical College of Wisconsin |
| **Janet Rader, MD, Professor of Obstetrics and Gynecology** | Jack A. & Elaine D. Klieger Professor and Chair of the Department of Obstetrics and Gynecology; Froedtert Hospital & Medical College of Wisconsin |

**Supplement 6: Institutional faculty and staff support**

| **Planning Phase** | # Meetings | Total Person Hours |
| --- | --- | --- |
| Planning Committee | 9 meetings | 171 hours |
| Leadership Advisory Committee | 2 meetings | 50 hours |
| Project Management & Ensemble Concept Training | 3 meetings | 21 hours |
| Project Management Training/Troubleshooting Office Hours | 31 meetings | 47 hours |
| **Initiative Rollout thru 04/30/2021** | # Meetings | Total Person Hours |
| Project Manager Support During Team Meetings | 370 meetings | 278 hours |
| Regular Status Report Meetings with Project Managers and CTSI Leadership (11:30 am and 4:30 meetings) | 135 meetings | 1,337 hours |

**Supplement 7: Team Status (As of 12/21/2020)**

|  | **Active** | **Retired** | **Merged** | **Subdivided** | **Dormant*** |
| --- | --- | --- | --- | --- | --- |
| # Teams | 14 | 15 | 7 | 3 | 2 |
| Average # Meetings | 19 | 5 | 2 | 2 | 8 |
| Median | 15 | 3 | 2 | 2 | 8 |

**Dormant = Waiting to determine whether to reactivate or retire the team*

**Supplement 8: Scholarly output for 12 teams that included either (1) grants (submitted or awarded), (2) publications, or (3) presentations.***

| **Team Unique Question** | **Grant Submissions and Awards** | **Publication or Presentation** |
| --- | --- | --- |
| What changes will COVID-19 bring to clinic workflow in post pandemic environment? | - Assisted Fast-track Follow-up from ED to Clinic Trial (AFFECT); Total Requested = $49,999 |  |
| Explore technologies that are in final stages of R&D to manage COVID-19 | - Total Cell-Free DNA as a Predictor of Severity of Illness and Outcome in Covid-19; Total Requested = $3,525,106. *Not Funded* |  |
| Collect feasibility data for conduct of future innovative Phase ½ clinical trials using existing drugs and biologics. | - **AWARDED: RAPID: How do vertebrate endolysosomal Ca2+ channels control coronaviral tropism? Total Requested = $199,909; Total Awarded = $199,909** - Efficacy and Safety of Oral N-Acetylcysteine in Outpatients with COVID-19: A Pilot Phase II Clinical Trial; Total Requested = $154,938. *Not Funded* - A Pilot Phase II, Placebo-Controlled, Double-Blind Study on Oral N-Acetylcysteine in Non-Hospitalized Patients with Mild Coronavirus Disease-2019; Total Requested = $363,151. *Not Funded* - A Phase II Randomized Controlled Trial of Salmeterol vs. Placebo for Inpatients with COVID-19 (The Salmeterol Trial); Total Requested = $8,384,478. *Not Funded* - A Phase II/III Randomized Controlled Trial of Salmeterol vs. Placebo for Outpatients with COVID-19 (The Salmeterol Outpatient Trial); Total Requested = $14,069,935. *Not Funded* | - **Publication:** Gunaratne GS, Brailoiu E, He S, Unterwald EM, Patel S, Slama JT, et al. Essential requirement for JPT2 in NAADP-evoked Ca(2+) signaling. Sci Signal. 2021;14(675).^44^ |
| Community responsive communications to inform at-risk Milwaukee populations about COVID-19 and how to survive, recover and thrive throughout the crisis. | - **AWARDED: Integrating Advocates Within a Healthcare Setting to Strengthen Intimate Partner Violence Screening; Total Requested = $384,979; Total Awarded = $384,979** - Designing and Implementing a Synergistic Community Model for Intimate Partner Violence; Total Requested = $2,033,003. *Not Funded* - Promoting Mental Health and Wellbeing of Vulnerable Milwaukee Communities Impacted by COVID-19; Total Requested = $50,000. *Not Funded* | - **Publication:** Galambos, Colleen PhD, ACSW, LCSW, FGSA, FAASWSW; Jerofke-Owen, Teresa PhD, RN; Paquette, Heidi PhD, RN, NNP-BC; Piacentine, Linda PhD, RN, ACNP-BC; Schubert, Erin PhD; Vang, Mychoua MPH; Arrington, Erica MD; Lodh, Nil PhD; Gecsi, Kimberly S. MD. Changing Intimate Partner Violence Screening and Intervention: Focus Group Perspectives. Journal of Nursing Care Quality 40(2):p 152-158, April/June 2025.DOI: 10.1097/NCQ.0000000000000823.^47^ - **Publication:** Sarawagi, A., Vang, M., Yan, K., Piacentine, L.B., Gecsi, K., Chelimsky, G., Galambos, C., Jerofke, T., Paquette, H., Schubert, E., & Lodh, N. Intimate Partner Violence Screening in an Obstetrics Clinic: A Retrospective Study. WMJ. 2024;123(6):441-445.^48^ - **Publication:** Schubert EC, Galambos CM, Jerofke-Owen T, Arrington E, Jordan GC, Lodh N, Paquette H, Chelimsky G, Piacentine LB. Building a community-academic partnership to improve screening for intimate partner violence: Integrating advocates in healthcare clinic settings. J Adv Nurs. 2023 Apr;79(4):1603-1609. doi: 10.1111/jan.15284. Epub 2022 May 27. PMID: 35621342.^46^ - **Presentation:** Lodh, N. et al. (2024, June 20). Intimate partner violence: Improving screening and referral, and integrating advocates in OB/GYN clinics (Ignite Style Presentation). Advancing Health for ALL Wisconsin Conference, Advancing a Healthier Wisconsin Endowment, Green Bay, WI.^49^ - **Presentation:** Jerofke-Owen, T., Paquette, H., Vang, M., Gecsi, K., Galambos, C., Schubert, E., Lodh, N. & Piacentine L.B. (2024, April 19). Intimate partner violence screening and referral in healthcare: A scoping review [Paper presentation]. 26th Annual Building Bridges to Research-Based Nursing Practice Southeastern Wisconsin Nursing Research Conference, Milwaukee, WI.^50^ - **Presentation:** Paquette, H., Piacentine L.B., Sarawagi, A., Vang, M., Yan, K., Gecsi, K., Chelimsky, G. Galambos, C., Jerofke-Owen, T., Schubert, E., & Lodh, N. (2024, April 19). Intimate partner violence screening in an obstetrics and gynecology clinic [Poster presentation]. 26th Annual Building Bridges to Research-Based Nursing Practice Southeastern Wisconsin Nursing Research Conference, Milwaukee, WI.^51^ - **Presentation:** Sarawagi, A., Vang, M., Yan, K., Piacentine, L., Gecsi, K., Chelimsky, G., Galambos, C., Jerofke, T., Paquette, H., Schubert, E., Lodh, N., (2024, March 7), Practices for Intimate Partner Violence Screening in an Obstetrics Clinic: A Retrospective Study [Virtual Conference Program abstract], 2024 Advancing Equity in the Learning Environment Summit: Joining Forces with Our Community and Healthcare Partners. Medical College of Wisconsin, Milwaukee, WI.^52^ - **Presentation:** Paquette, H., Schubert, E., Galambos, C., Jerofke-Owen, T., Arrington, E., Jordan, G., Lodh, N., Chelimsky, G., Piacentine, L., & Gecsi, K. (2022, November 3). Building a Community-Academic Partnership to Improve Screening for Intimate Partner Violence: Integrating Advocates in Healthcare Clinic Settings [Poster presentation]. Medical College of Wisconsin Community Engagement Poster Session, Milwaukee, WI.^53^ |
| Can we improve COVID-19 PPE effectiveness and sterilization, durability? | - Integrity of N95 Respirators Through Extended Use and Decontamination Cycles; Total Requested = $22,797. *Not Funded* - Integrity of N95 Respirators Through Extended Use and Decontamination Cycles; Total Requested = $49,958. *Not Funded* |  |
| Building Resilience Virtually (BRaVe) Clinic | - **AWARDED: Psychological Sequelae of Healthcare Workers; Total Requested = $43,730; Total Awarded = $25,000** | - **Publication:** Kroll KH, Larsen S, Lamb K, Davies WH, Cipriano D, deRoon-Cassini TA, et al. Responding to the Psychological Needs of Health-Care Workers During the COVID-19 Pandemic: Case Study from the Medical College of Wisconsin. J Clin Psychol Med Settings. 2022;29(1):150-61.^58^ |
| Develop COVID-19 Diagnostic Tools. | - Deep Learning-based Image-biotechnology Sensor for Rapid Diagnostic Test of SARS-CoV-2 in Oral Fluids for Healthcare Providers; Total Requested = $460,691. *Not Funded* |  |
| Generate timely evidence on the direction and magnitude of association between the use of ACEI or ARBs and COVID-19 severity and mortality. |  | - **Publication:** Cain MT, Smith NJ, Barash M, Simpson P, Durham LA, 3rd, Makker H, et al. Extracorporeal Membrane Oxygenation with Right Ventricular Assist Device for COVID-19 ARDS. J Surg Res. 2021;264:81-9.^42^ - **Publication:** Cain MT, Taylor LJ, Colborn K, Teman NR, Hoffman J, Mayer KP, Etchill EW, Sevin CM, Jaishankar S, Ramanan R, Enfield K, Zwischenberger JB, Jolley SE, Rove JY; ORACLE group. Worse survival in patients with right ventricular dysfunction and COVID-19-associated acute respiratory distress requiring extracorporeal membrane oxygenation: A multicenter study from the ORACLE Group. J Thorac Cardiovasc Surg. 2024 May;167(5):1833-1841.e2. doi: 10.1016/j.jtcvs.2022.12.013. Epub 2022 Dec 21. PMID: 36717346; PMCID: PMC9767877.^45^ - **Publication:** Smith NJ, Park S, Zundel MT, Dong H, Szabo A, Cain MT, Durham LA 3rd. Extracorporeal membrane oxygenation for COVID-19: An evolving experience through multiple waves. Artif Organs. 2022 Nov;46(11):2257-2265. doi: 10.1111/aor.14381. Epub 2022 Aug 20. PMID: 35957490; PMCID: PMC9538401.^43^ - **Presentation:** Cain MT SN, Barash M, Simpson P, Durham LA, 3rd, Makker H, Roberts C, Falcucci, O, Wang D, Walker R, Ahmed G, Brown S, Nanchal RS, Joyce DL. Extracorporeal Membrane Oxygenation with Right Ventricular Assist Device for COVID-19 ARDS. 16th Annual Academic Surgical Congress: A Virtual Experience; 2021 February 2-4, 2021; Virtual: Association for Academic Surgery.^56^ |
| Develop Point of Care testing for COVID-19 and other infectious diseases. | - Rapid, Easy to Use, Dendrimer Nanotechnology based COVID-19 Combo Antibody-Antigen Detection Kit for Home and Low Resources Setting; Total Requested = $429,054. *Not Funded* |  |
| Study life history of COVID-19 to develop better predictive models of spread, transmissibility and interventions. | - Health Radius (Title not available); Total Requested = $50,000. *Not Funded* - Health Radius (Title not available); Total Requested = $300,000. *Not Funded* |  |
| What are the psychological sequelae from COVID-19? | - Addressing Psychosocial Support for Wisconsin’s Caregivers of Children with Special Needs; Total Requested = $50,000. *Not Funded* - CTSI Pilot Grant: COVID-19 Impact on Psychological Wellbeing of Caregivers in Response to Increased Caregiver Stress; Total Requested = $35,000. *Not Funded* | - **Presentation:** Beverung JS, Laura Brusky, Colleen Galambos, W. Hobart Davies, Abiola Keller, Abir Bekhet, Robyn Hardt Schultz, Julia Ann Hyatt, Louann Sullivan. Impact of COVID-19 on Caregiver Stress. 2021 Association for Psychological Science Virtual Convention; May 26; Virtual 2021. p. 207.^54^ - **Presentation:** Beverung, L. M., Schnell, J., Brusky, L., Galambos, C., Davies, W.H., Keller, A., Bekhet, A., Hardt Schultz, R., & Sullivan, L. [May 2022]. Family Caregiving during a Pandemic: Well-Being Is Multifaceted. Association for Psychological Science Annual Convention, Chicago.^55^ |
| Substance use changes in drug and alcohol use during COVID-19. | - Predicting COVID-19 Outcomes for Individuals with Substance Use Disorder; Total Requested = $1,470,515. *Not Funded* - Changes in Drug and Alcohol Abuse during the COVID-19 Crisis; Total Requested = $401,054. *Not Funded* |  |

*The remaining teams did not have scholarly output. However, some of the remaining teams may have developed projects with a shared vision, specific aims, recruited team expertise, identified resources, or other preparation, but these teams did not go beyond that stage.
